# Mapping the global distribution of invasive pest *Drosophila suzukii* and parasitoid *Leptopilina japonica*: implications for biological control

**DOI:** 10.7717/peerj.15222

**Published:** 2023-04-24

**Authors:** Rahul R. Nair, A. Townsend Peterson

**Affiliations:** Biodiversity Institute, University of Kansas, Lawrence, KS, United States of America

**Keywords:** *Drosophila suzukii*, *Leptopilina japonica*, Pest, Parasitoid, Invasion, Biological control, Ecological niche modeling

## Abstract

Insect pest invasions cause significant damage to crop yields, and the resultant economic losses are truly alarming. Climate change and trade liberalization have opened new ways of pest invasions. Given the consumer preference towards organic agricultural products and environment-friendly nature of natural pest control strategies, biological control is considered to be one of the potential options for managing invasive insect pests. *Drosophila suzukii* (Drosophilidae) is an extremely damaging fruit pest, demanding development of effective and sustainable biological control strategies. In this study, we assessed the potential of the parasitoid *Leptopilina japonica* (Figitidae) as a biocontrol agent for *D. suzukii* using ecological niche modeling approaches. We developed global-scale models for both pest and parasitoid to identify four components necessary to derive a niche based, target oriented prioritization approach to plan biological control programs for *D. suzukii*: (i) potential distribution of pest *D. suzukii*, (ii) potential distribution of parasitoid *L. japonica*, (iii) the degree of overlap in potential distributions of pest and parasitoid, and (iv) biocontrol potential of this system for each country. Overlapping suitable areas of pest and parasitoid were identified at two different thresholds and at the most desirable threshold (*E* = 5%), potential for *L. japonica* mediated biocontrol management existed in 125 countries covering 1.87 × 10^7^ km^2^, and at the maximum permitted threshold (*E* = 10%), land coverage was reduced to 1.44 × 10^7^ km^2^ in 121 countries. Fly pest distributional information as a predictor variable was not found to be improving parasitoid model performance, and globally, only in half of the countries, >50% biocontrol coverage was estimated. We therefore suggest that niche specificities of both pest and parasitoid must be included in site-specific release planning of *L. japonica* for effective biocontrol management aimed at *D. suzukii*. This study can be extended to design cost-effective pre-assessment strategies for implementing any biological control management program.

## Introduction

Over recent decades, the world has witnessed significant increases in agricultural production, but increases in crop yields have often been reduced by diverse insect pests (Vreysen et al., 2007; Savary et al., 2019). Assessment of all of the components of agricultural productivity and food security must include consideration of insect pests, as they are an integral part of anthropogenic crop ecosystems (Food and Agriculture Organization, 2013; Savary et al., 2019). Global warming and economic globalization accelerate development of new routes of pest invasion (Girod et al., 2018), presenting new challenges. As pests pose serious threats in the functioning of global food systems (Savary et al., 2017), various strategies have been developed for insect pest management, each with its own advantages and disadvantages (Dara, 2021). Improvement in the management of invasive pest populations includes consideration of sustainable and eco-friendly approaches, with the goal of achieving long-term benefits (Bernaola & Holt, 2021).

A broad (fruit) host range (Lee et al., 2011; Bellamy, Sisterson & Walse, 2013), combined with an ability to infest ripening soft fruits (Gabarra et al., 2015), has made *Drosophila suzukii* (Matsumura) (Diptera: Drosophilidae) an economically damaging, globally invasive fruit pest of serious concern (Walsh et al., 2011). Preference for not-quite-ripe or just-ripe fruits over damaged or decaying fruits (Mitsui, Takahashi & Kimura, 2006), and the presence of a sclerotized ovipositor of females (Kienzle & Rohlfs, 2021) with serrations to pierce undamaged fruit epicarps for laying eggs, are two notable traits (Walsh et al., 2011) that contribute significantly to economic threats imposed by *D. suzukii*. Bacterial and fungal pathogens can cause secondary infections in fruits after infestation by *D. suzukii,* augmenting economic losses (Molina, Harrison & Brewer, 1974; Louis et al., 1996; Walsh et al., 2011).

*Drosophila suzukii* is native to eastern and southeastern Asia (Bolda, Goodhue & Zalom, 2010); it was initially detected in Japan in 1916 (Kanzawa, 1935) and described as a distinct species in 1931 (Hauser, 2011). In 2008, *D. suzukii* was identified as an invasive species for the first time with populations in both North America (Hauser, 2011) and Europe (Calabria et al., 2012). Its host range covers 13 angiosperm families (Cloonan et al., 2018), and its invaded geographic range has now extended to South America (Deprá et al., 2014; Andreazza et al., 2017) and Africa (Kwadha et al., 2021). As *D. suzukii* larvae feed inside of fruits (Fanning, Grieshop & Isaacs, 2018), and the fruit export trade strictly follows zero-tolerance towards infestations (Tait et al., 2021), much high-value fruit is rendered unsellable every year. Economic impact assessments in the United States (Bolda, Goodhue & Zalom, 2010; Walsh et al., 2011; Goodhue et al., 2011; Farnsworth et al., 2017; DiGiacomo et al., 2019; Yeh et al., 2020), Europe (Knapp, Mazzi & Finger, 2021), and South America (Benito, Lopes-da Silva & Santos, 2016), have indicated losses on the order of US$550M per year.

Various preventive and post-infestation control measures (Lee et al., 2011; Landolt, Adams & Rogg, 2012; Haye et al., 2016; Schetelig et al., 2018; Shawer et al., 2018; Tait et al., 2021) have been developed so far, but none with complete efficacy (Kehrli et al., 2017; Knapp, Mazzi & Finger, 2019). Management strategies for *D. suzukii* can be classified broadly into four categories: (1) chemical control (Beers et al., 2011; Van Timmeren & Isaacs, 2013; Shawer et al., 2018; Shawer, 2020), (2) microclimate manipulation (Lee et al., 2016; Rendon et al., 2020), (3) RNA interference biopesticides (Murphy et al., 2016), and (4) biological control (Chabert et al., 2012; Daane et al., 2016; Mazzetto et al., 2016; Knoll et al., 2017; Daane et al., 2021). Extensive use of chemical methods to control *D. suzukii* infestations can lead to increased pest resistance, and concerns regarding food and environmental safety (Santoiemma et al., 2019). Microclimate manipulation approaches to control *D. suzukii* are more likely to perform well in hot and dry regions (Schöneberg et al., 2022), as *D. suzukii* is sensitive to high temperatures and low humidity (Rendon et al., 2020). RNA interference methods involve higher development costs and involve much labor (Bramlett, Plaetinck & Maienfisch, 2020). Finally, biological control involves release of enemies of *D. suzukii* from the region of its origin (Asia) in invaded areas, as a means to reduce its population growth (Girod et al., 2018). This method is recommended (Cock et al., 2010; Van Lenteren, 2012) in view of improved food safety, environment-friendly characteristics, economic feasibility, and long-term control solutions that are established (Kruitwagen, Beukeboom & Wertheim, 2018).

Parasitoid wasps of the genera *Asobara* (Braconidae), *Ganaspis* (Figitidae), and *Leptopilina* (Figitidae) have been studied extensively as biological control agents with potential to suppress growth of *D. suzukii* populations (Kacsoh & Schlenke, 2012; Rossi Stacconi et al., 2015; Daane et al., 2016; Giorgini et al., 2019; Wang et al., 2019; Biondi, Wang & Daane, 2021). In particular, the species *A. japonica*, *G. brasiliensis*, and *L. japonica* are potential biocontrol agents (Wang et al., 2019). However, some researchers do not recommend *A. japonica* for biological control programs aimed at *D. suzukii* (Daane et al., 2016; Girod et al., 2018; Abram et al., 2020), owing to its broad host range (Ideo et al., 2008; Furihata et al., 2016). Indeed, given its host specificity, *G. brasiliensis* has been proposed as a candidate for biological control of *D. suzukii* (Wang et al., 2020); yet, in a scenario when these three wasps coexist, *L. japonica* is unique in being able to outcompete the other two species thanks to its relatively faster egg-hatching potential (Wang et al., 2019). Relatively high host specificity (Wang et al., 2020), demonstrated competence in multi-parasite systems (Wang et al., 2019), and recent range expansions into areas invaded by *D. suzukii* in Europe (Puppato et al., 2020) and North America (Abram et al., 2020; Abram et al., 2022), make *L. japonica* an intriguing candidate biocontrol agent for *D. suzukii* that can be tested for overall effectiveness.

Ecological niche modeling (ENM) has been used extensively to identify potential distributions of species for a variety of purposes (Raxworthy et al., 2007; Escobar, 2020; Kolanowska & Jakubska-Busse, 2020; Wan et al., 2020; Valencia-Rodríguez et al., 2021; Agboka et al., 2022; Demján et al., 2022; Outammassine, Zouhair & Loqman, 2022). In pest-parasitoid systems, identifying and comparing relative habitat suitability of pest and parasitoid can help to guide effective biological control programs (Pérez-de la O et al., 2020; Tepa-Yotto et al., 2021a; Tepa-Yotto et al., 2021b). The utility of ENM in applications to biological control of pests can be attributed to two factors: alien parasitoid species must survive and reproduce in the geographic regions where they are released (Mills, 2018; Schulz, Lucardi & Marsico, 2019), and unfavorable abiotic factors can reduce the long-term efficacy of biological control measures (Olfert et al., 2016). Modeling climatic preferences of deliberately introduced parasitoid species can also provide insights into possible range expansions, an important aspect to be tested in improving effectiveness of classical biological control programs (Pérez-de la O et al., 2020).

In this study, we used ENM approaches to explore, discuss, and highlight five aspects of a biological control strategy for *D. suzukii* that can directly benefit producers, extension agents, and policy makers. (1) We estimated the potential distribution of the invasive pest *D. suzukii*, and (2) that of the parasitoid *L. japonica*. (3) We assessed the degree of overlap in the potential distributions of *D. suzukii* and *L. japonica*, and (4) estimated the biocontrol potential of this system for each country. Finally, (5) we assessed parasitoid model performance to see if incorporating distributional information for the pest improves model performance for the parasitoid.

## Methods

### Occurrence data

Occurrence records of *D. suzukii* were downloaded from five online biodiversity data portals: Global Biodiversity Information Facility (GBIF; http://www.gbif.org, accessed on 2 August, 2022; DOI: https://doi.org/10.15468/dl.hxg8z2), Biodiversity Information Serving Our Nation (BISON; http://www.gbif.us, accessed on 2 August, 2022), Berkeley Ecoinformatics Engine (Ecoengine; ecoengine.berkeley.edu, accessed on 2 August, 2022), iNaturalist (http://www.inaturalist.org, accessed on 2 August, 2022), and Integrated Digitized Biocollections (iDigBio; http://www.idigbio.org, accessed on 2 August, 2022) using Spocc version 1.2.0 R package (Chamberlain, Ram & Hart, 2021); occurrence data were also drawn from the Centre for Agriculture and Bioscience International (CABI; http://www.cabi.org, accessed on 3 August, 2022), and published literature (see File S1 for details). This initial harvest of occurrence data yielded an initial total of 2369 records.

**Figure 1:**
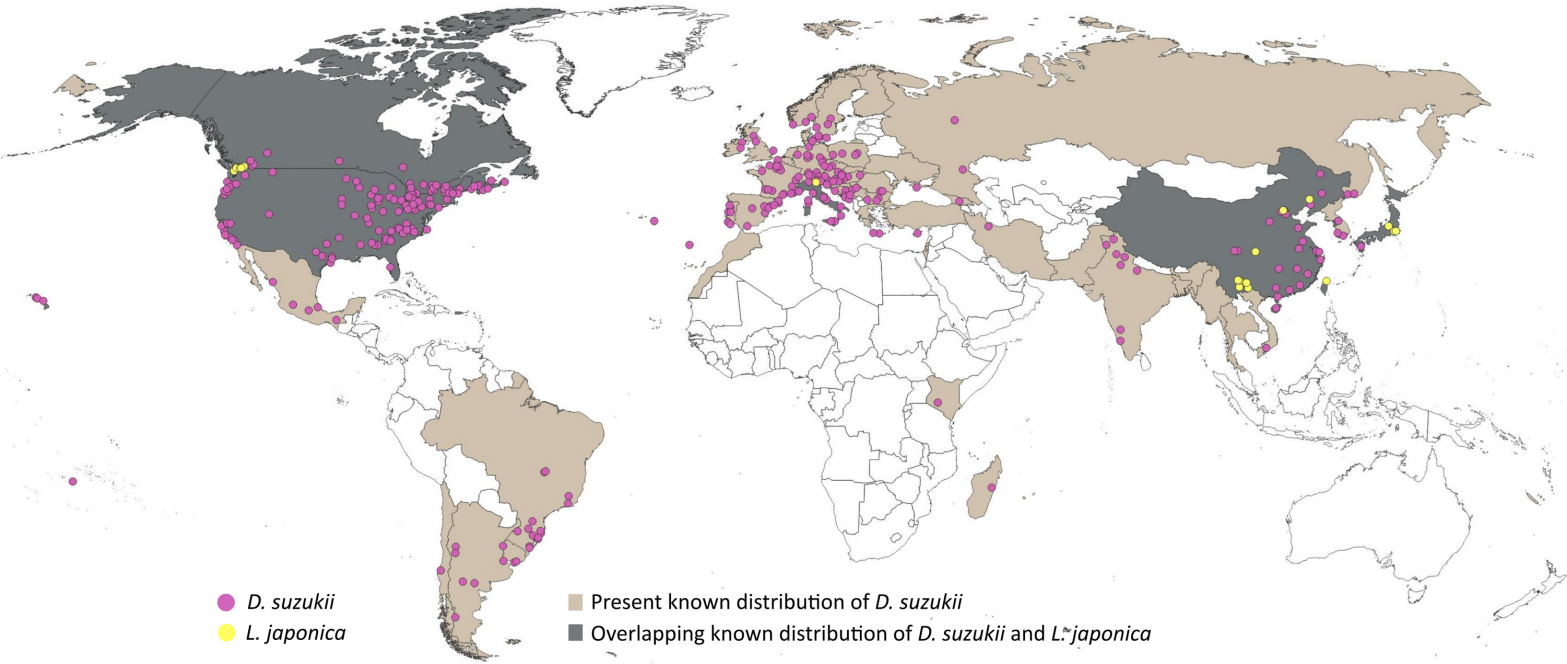
Distributional information. Representation of the known distribution of the pest *Drosophila suzukii*, and parasitoid *Leptopilina japonica* based on occurrence databases and published literature.

A five-step data cleaning process was adopted: (1) removal of records with no date of observation, (2) removal of incomplete coordinates (*i.e*., lacking valid latitude and longitude), (3) removal of unlikely coordinates (*e.g.*, 0.00°N, 0.00°E), (4) removal of duplicated coordinates, and (5) removal of coordinates with fewer than two decimal places. Data cleaning was performed using scrubr version 0.1.1 R package (Chamberlain, 2016). The cleaned dataset (1385 records) was overlaid on climatic raster layers (5′ or ∼10 km spatial resolution, see below) to remove points falling outside the raster boundaries. The resulting occurrence dataset (1377 records) was subjected to visual inspection to detect clusters of points (often related to points of access or concentrations of people), and eliminate disproportionate data density at random, maintaining a minimum distance of ≥ 30km among points, to avoid model overfitting (Raghavan et al., 2019). The final dataset of 314 points (Fig. 1; File S1) showed no excessive clustering of occurrences across the known distribution of *D. suzukii.* Spatial filtering was performed using spThin R package (Aiello-Lammens et al., 2015).

Occurrence records of *L. japonica* were sourced from published literature (Abram et al., 2022; Wang et al., 2022; Abram et al., 2020; Puppato et al., 2020; Giorgini et al., 2019; Girod, 2018; Novković et al., 2011), as online data portals held few or no records. A distance filter of 12 km was applied to the occurrences extracted, and the final dataset comprised 31 points (Fig. 1; File S2). *Leptopilina japonica* has two subspecies: *L. japonica japonica* and *L. japonica formosana*, occurring in Japan and Taiwan respectively (Novković et al., 2011); both have the ability to parasitize *D. suzukii* (Kimura & Novković, 2015). Our final dataset included mostly the nominate subspecies, and only a single occurrence record of *L. j. formosana* (Novković et al., 2011).

### Environmental data

Bioclimatic raster layers at 5′ spatial resolution (∼10 km at the Equator) were downloaded from WorldClim 2.1 for present conditions (1975–2000; Fick & Hijmans, 2017). Variables combining temperature and precipitation measurements (*i.e*., mean temperature of wettest quarter, mean temperature of driest quarter, precipitation of warmest quarter, and precipitation of coldest quarter) were excluded (Escobar et al., 2014) owing to discontinuous patterns of those variables in many areas (Booth, 2022).

To define the set of limits and conditions for ENM, identification of areas accessible to species over relevant time periods (Soberón & Peterson, 2005; Peterson & Soberón, 2012) is essential to development of robust models (Barve et al., 2011). The development of a hypothesis of accessible area **M** is crucial for rigorous characterization of niche characteristics of species (Barve et al., 2011; Machado-Stredel, Cobos & Peterson, 2021). Considering the near-global distribution of *D. suzukii* and *L. japonica,* the entire world (excluding Antarctica) was defined as the accessible area for the two species. The 15 climatic data layers were clipped to the extent of this area. Multi-collinearity and dimensionality among the clipped bioclimatic layers were minimized using principal components analysis, in effect transforming correlated climatic variables into fewer, uncorrelated principal components (PCs), and these multivariate environmental variables were used as the independent variables in ENM.

The advantage of principal component analysis over other methods of multi-collinearity reduction is that a significant proportion of all original information related to variables can be retained in the form of independent components (Tabachnick & Fidell, 2007; Cruz-Cárdenas et al., 2014), summarizing all environmental variation across a particular geographic region (Júnior & Nóbrega, 2018). Each PC is a linear combination of all of the 15 original climatic variables: the first PC summarizes the major axis of the multivariate space, explaining a large proportion of the total variance in the original data; the second PC explains a maximum of the remaining variance, which is independent of the first axis; and so on (Cruz-Cárdenas et al., 2014). Principal components analysis of raster variables was done using the kuenm_rpca function of the kuenm R package (Cobos et al., 2019). Contributions of each of the original bioclimatic variables to the PCs (Sillero et al., 2021) and average contribution of each of the PCs to the final models of pest and parasitoid were estimated (Quiner & Nakazawa, 2017), to have insight into important variables driving niches and distributions of pest and parasitoid. We applied an arbitrary threshold of absolute value of factor loadings to assess the relative importance of variables to each of the PCs (Rotenberry, Preston & Knick, 2006; Barrows et al., 2008); variables with factor loadings ≥0.35 were explored as potentially important (Bogosian III et al., 2012). Loading values of the variables represent the extent to which those variables are correlated with particular PCs (Júnior & Nóbrega, 2018). In the case of PCs with mixed positive and negative loadings, variables with positive loadings >0.35 contribute the same amount of information as that of variables with negative loading <−0.35, as, in both cases, the absolute value of loadings exceeds our arbitrary threshold of 0.35. The signs of the loadings indicate the nature of correlation of variables with the PCs.

### Ecological niche modeling

In separate ENM analyses, occurrences of each species (pest and parasitoid) were partitioned randomly into training and testing data in two different proportions: 70:30 for *D. suzukii*, and 50:50 for *L. japonica*. Considering the small number of records, data-splitting ratio was reduced to 0.5 for *L. japonica* to maintain a balance between predictive accuracy and performance estimation of models as very low sample size for testing can cause errors in estimating predictive accuracy (Peterson, Ball & Cohoon, 2002). Modeling experiments were performed using six combinations of three feature classes (l-linear, q-quadratic, p-product; l, q, lq, qp, lp, and lqp; product response types were not used in isolation owing to occasional problems that result), 10 regularization multipliers (0.1, 0.3, 0.6, 0.9, 1, 2, 3, 4, 5, 6), and nine sets of principal components summarizing climate data. The first 10 PCs accounted for >99% of the total variation: set 1 (PCs 1 and 2), set 2 (PCs 1-3), *etc*., up to set 9 (PCs 1-10). Best models were selected by applying three criteria sequentially (Cobos et al., 2019): (1) choosing statistically significant models using partial ROC tests, (2) filtering statistically significant models to those with <5% omission error (*E*), and (3) ranking all remaining models based on Akaike information criterion (AICc) values; the subset of significant, low-omission models within 2 AICc units of the minimum were selected as the best models (Warren & Seifert, 2011).

Mean AUC ratios of bootstrap replicate models were calculated using the partial ROC approach, which remedies some of the known problems with traditional receiver operating curve (ROC) analysis (Peterson, Papeş & Soberón, 2008). In this method, the importance of negative (absence) information is reduced, as such information is generally unavailable (Peterson, Papeş & Soberón, 2008). Crucially, the interpretation of the area under the curve is limited to relevant portions of the curve, that is those parts meeting user-defined low omission thresholds, in this study *E* = 5%. Then, as one is generally not evaluating the curves over the entire space, to assess statistical significance, AUC ratios are defined as the ratio of AUC of the partial ROC curve to the area under the random expectation line over the same restricted part of the space. AUC ratio values range from 0 to 2; a value of 1 indicates random performance (Peterson, Papeş & Soberón, 2008; Peterson, 2012). Model fitting was replicated 10 times using bootstrapped subsamples of the available occurrence data; variation among replicates was then used to assess whether the AUC ratio exceeds 1 significantly, and the median of the median suitability outputs across all replicates was used to interpret results for each species.

To assess the potential role of fly distributional information in improving the performance of the parasitoid model, the final *D. suzukii* model output was added to each multivariate environmental variable set. We then re-calibrated the *L. japonica* model using the same set of feature class types and regularization multiplier values to develop a two-species model for the wasp (see Ashraf, Chaudhry & Peterson, 2021). We compared models with and without the fly distributional information using the same 3 criteria described above. Occurrence data partitioning exercises were done using caTools R package (Tuszynski, 2021). All modeling experiments were performed using maximum entropy approaches (Maxent) (Phillips, Anderson & Schapire, 2006), as implemented in the kuenm R package (Cobos et al., 2019).

To represent suitable and unsuitable regions for the pest and the parasitoid, Maxent models in the form of continuous logistic outputs were transformed into binary presence-absence models by applying two different least-training presence thresholds (*i.e*., allowable omission *E* = 5% and *E* = 10%). These two thresholds were chosen as indices of most desirable (*E* = 5%) and maximum permitted (*E* = 10%) omission rates to represent relative habitat suitability, and also to avoid overinterpretation of predictions (Ashraf, Chaudhry & Peterson, 2021). These thresholds were applied using QGIS Tisler desktop version 3.24.3 (QGIS Geographic Information System, 2022).

Similarity between niche estimates for pest and parasitoid was quantified using Schoener’s *D* index based on two methods: an ENM-based method which compares niches in geographic space (Warren, Glor & Turelli, 2008) and a parallel, ordination-based method (PCA-env) that compares niches in environmental space using similar tests (Broennimann et al., 2012). Given that the area **M** was same for both pest and parasitoid, a symmetric background similarity test (Warren, Glor & Turelli, 2008; Warren et al., 2021) was used to implement the ENM-based method. The observed *D* value of the two empirical models (pest and parasitoid) was compared with a null distribution of D values generated by comparing the expected overlap of the niche estimates of 100 replicates of pest and parasitoid models, developed by drawing random occurrences 100 times from the background of both species, retaining the original sample sizes (Warren, Glor & Turelli, 2008; Warren et al., 2021). In environmental space, the PCA-env method (Broennimann et al., 2012) was used to summarize climatic variability across the **M** area of both species. This method tests whether the niche occupied by pest is similar to that occupied by parasitoid. Occurrence densities of pest and parasitoid were shifted randomly 100 times in the background, and niche overlap was calculated in each iteration to create a null distribution of *D* values. In both cases, we used a one-tailed test focusing on rejecting a null hypothesis of niche similarity (Peterson, 2011; Tocchio et al., 2015; Qiao, Escobar & Peterson, 2017), and ignoring the upper tail of the distribution that would be significant niche similarity, which is of unknown biological meaning. Non-rejection of the null hypothesis of niche similarity arises when the empirical D value falls within the upper 95% of the null distribution of D values (*P* > 0.05) (Tocchio et al., 2015), indicating that niches of the two species are not demonstrably distinguishable. We used the ENMTools 1.0 (Warren et al., 2021) and ecospat (Di Cola et al., 2017) R packages to implement the niche similarity tests.

For both thresholds, overlapping potential habitats of *D. suzukii* and *L. japonica* were identified. The ratio between the land areas of predicted potential distribution of parasitoid and pest in each country was estimated to determine the country-wise biocontrol coverage potential percentage, for both thresholds. Identification of overlapped area and estimation of land area in terms of biocontrol coverage were done in QGIS Tisler desktop version 3.24.3 (QGIS Geographic Information System, 2022). All models were represented in an Eckert III map projection.

## Results

For each of the two species, we developed 540 candidate models, of which 510 models for *D. suzukii* and 533 models for *L. japonica* were statistically significantly better than random expectations according to the partial ROC tests (*P* < 0.05). Of the statistically significant models, 53 models for *D. suzukii* and 11 models for *L. japonica* were also acceptable in having low (<5%) omission. Finally, based on low model complexity (*i.e*., low AICc value), our top model for *D. suzukii* included linear and quadratic feature classes, a relatively low regularization multiplier value (0.6), and four multivariate environmental variables (PC 1–PC 4) (Table 1). Our best model for *L. japonica* had a higher regularization multiplier value (2.0), and included more multivariate environmental variables (PC 1–PC 7), also with linear and quadratic feature types (Table 1). In the two-species modeling experiment, we developed 540 models, and all models were statistically significantly better than random expectations (*P* < 0.05). However, none of the models met the omission rate threshold (*E* = 5%). We found that, even relaxing the threshold (*E* = 7%) did not result in the selection of any of the two-species models as best model for parasitoid. We therefore confirmed that inclusion of pest model as a predictor variable did not improve model performance for the parasitoid.

**Table 1:** Model evaluation. Performance summary of pest, parasitoid, and two-species parasitoid models.

**Species**	**Models**	**Mean AUC ratio**	**OR**	**AICc**
*Leptopilina japonica*	M_2.0_F_lp_Set_6	1.76	0.00	839.55
*Drosophila suzukii*	M_0.6_F_lq_Set_3	1.47	0.04	8365.78
Two-species	M_1.0_F_l_Set_5	1.79	0.07	798.08

**Notes.**

OR-Omission rate. Name of models indicates the details of regularization multiplier value, feature class and environmental dataset.

Collective contributions of PC1 and PC2 to the final model for *D. suzukii*, and that of PC1, PC3, and PC7 to the final model for *L. japonica* was >75% (Table 2). For PC1 in the *D. suzukii* model, no individual climatic variables met the factor loading criterion of 0.35. However, on PC2, variables meeting that criterion included a contrast of precipitation of driest quarter (0.41) and precipitation during driest month (0.40) with mean diurnal range (−0.38). Other variables that fell just short of the threshold were mean temperature of coldest quarter (0.34) and minimum temperature of coldest month (0.34). For the *L. japonica* model, isothermality (0.82) was the largest contributor to PC7, and PC3 was a contrast of precipitation of wettest quarter (0.40), precipitation during wettest month (0.43), and precipitation seasonality (0.52) with precipitation of driest month (−0.35). Mean diurnal range (−0.34) fell just short of the threshold.

**Table 2:** Important variables. Relative contribution of climatic variables to principal components.

**Variables**	**Principal components**						
	PC1	PC2	PC3	PC4	PC5	PC6	PC7
Annual mean temperature	0.33	−0.17	−0.12	0.01	0.15	−0.07	−0.07
Mean diurnal range	0.10	**−0.38**	−0.14	0.34	**−0.58**	**0.47**	−0.34
Isothermality	0.32	−0.02	0.00	−0.14	**−0.37**	0.18	**0.82**
Temperature seasonality	−0.32	−0.02	−0.01	**0.39**	0.15	−0.07	0.29
Maximum temperature of warmest month	0.25	−0.29	−0.20	0.32	0.27	−0.06	0.10
Minimum temperature of coldest month	0.34	−0.08	−0.09	−0.17	0.12	−0.06	−0.08
Temperature annual range	−0.30	−0.11	−0.02	**0.48**	0.04	0.04	0.19
Mean temperature of warmest quarter	0.27	−0.26	−0.19	0.25	0.33	−0.16	0.10
Mean temperature of coldest quarter	0.34	−0.11	−0.08	−0.13	0.06	−0.03	−0.13
Annual precipitation	0.24	0.34	0.15	0.22	0.03	0.13	0.11
Precipitation of wettest month	0.25	0.22	**0.43**	0.24	0.13	0.16	−0.13
Precipitation of driest month	0.12	**0.40**	**−0.35**	0.21	−0.25	−0.31	−0.10
Precipitation seasonality	0.09	−0.31	**0.52**	0.11	−**0.38**	−**0.68**	−0.02
Precipitation of wettest quarter	0.25	0.24	**0.40**	0.24	0.12	0.19	−0.08
Precipitation of driest quarter	0.13	**0.41**	−0.33	0.21	−0.22	−0.26	−0.05
Average contribution of PCs to final model of pest	56.62	36.63	6.49	0.27	–	–	–
Average contribution of PCs to final model of parasitoid	10.81	2.14	13.42	4.41	6.58	8.07	54.57

**Notes.**

Factor loadings ≥ 0.35 are shown in bold.

**Figure 2:**
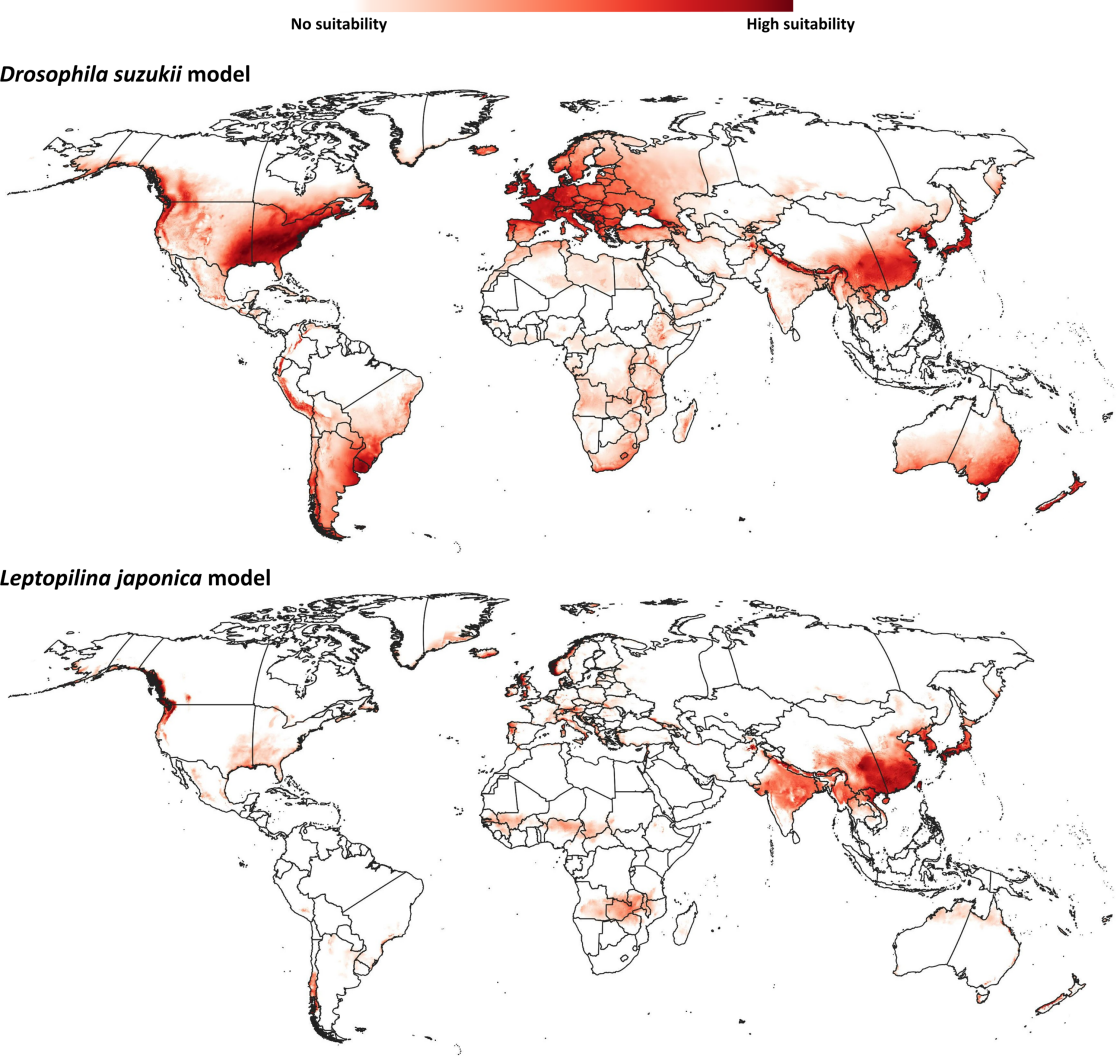
Ecological niche models. Predicted distribution of potential distributional areas of *Drosophila suzukii* and *Leptopilina japonica* across the world.

Our model for *D. suzukii* predicted potential distributional areas in southern and eastern China, with some extensions towards central Asian regions (Fig. 2). Farther north in Asia, Japan and the Korean Peninsula were predicted to hold broad suitable areas for *D. suzukii*. Predicted suitable areas covered seven nations [Afghanistan, Pakistan, India, Nepal, China (Tibetan Autonomous Region), Bhutan, and Myanmar] across the entire northwest-southeast spread of the Himalayas. In Oceania, southeastern Australia and much of New Zealand were predicted to hold suitable conditions for *D. suzukii* invasion.

Already-invaded parts of western Europe and the southeastern United States were identified as highly suitable for *D. suzukii* populations, which is logical given that occurrences there were part of the model training data. In South America, the entire geographic extent of Uruguay, known to hold invasive populations, was identified as suitable for *D. suzukii*; parts of other known-invaded countries (Chile, Argentina, Brazil) were also identified as suitable: eastern and northeastern Argentina, southern Brazil, and western and southern Chile. Peru is the only country in South America predicted to hold suitable areas for *D. suzukii* invasion for which no invasive populations are known; predicted potential distributional areas spanned the Andean Cordillera.

The modeled potential geographic distribution for *L. japonica* (Fig. 2) was broad and continuous in Asia, covering southern and northeastern Asian countries (India, China (Tibetan autonomous region), Nepal, Bhutan, North Korea, South Korea, and Japan). Other potential distributional areas were more sparse, in northwestern Europe, western North America, and in western and southern Chile in South America.

### Binary models and biocontrol coverage estimation

**Figure 3:**
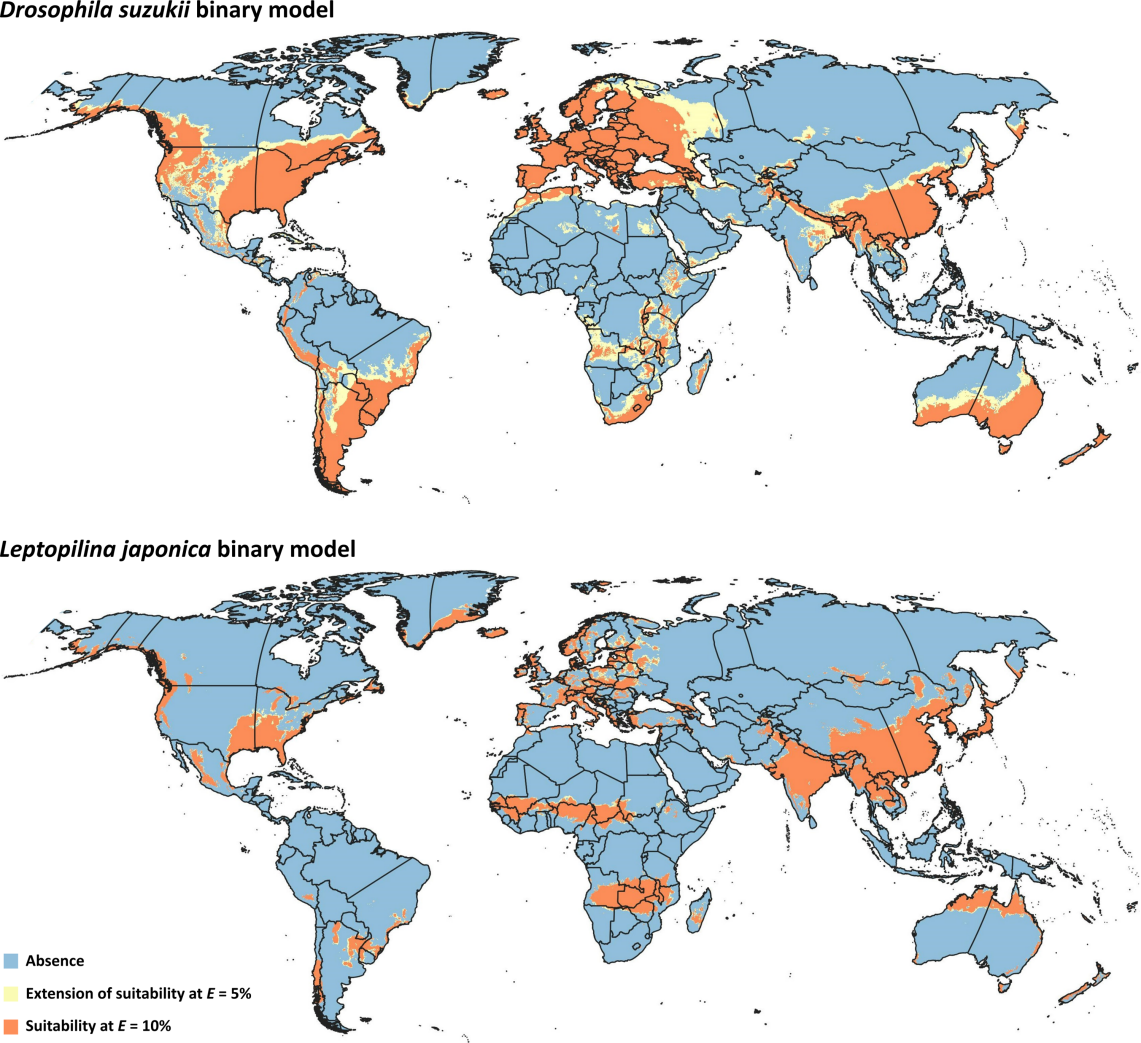
Binary models. Modeled suitable areas for *Drosophila suzukii* and *Leptopilina japonica* based on thresholding at *E* = 5% and *E* = 10%.

Binary model outputs were developed for *D. suzukii* and *L. japonica* (Fig. 3) to identify presence or absence of the two species in the area of interest. At the 5% threshold, potential presence of *D. suzukii* was predicted in 162 countries (File S3), covering a total area of ∼4.82 × 10^7^ km^2^. Potential presence of *L. japonica* was predicted in 148 countries (File S3), covering a total area of 2.71 × 10^7^ km^2^. At the 10% threshold, total coverage of predicted area was reduced to 3.44 × 10^7^ km^2^ in 152 countries for *D. suzukii*, and 2.46 × 10^7^ km^2^ in 146 countries for *L. japonica* (File S3).

Niches of pest and parasitoid were not demonstrably distinct in either geographic (empirical *D* = 0.76, *P* > 0.05) or environmental (empirical *D* = 0.35, *P* > 0.05) spaces, as the observed *D* values fell within the 95% confidence limits of the null distribution of *D* values in both methods (File S4). As such, no empirical evidence indicates that the two species have distinct ecological niches, and their distributional overlap can be explored as a bellweather of potential for distributional co-occurrence.

**Figure 4:**
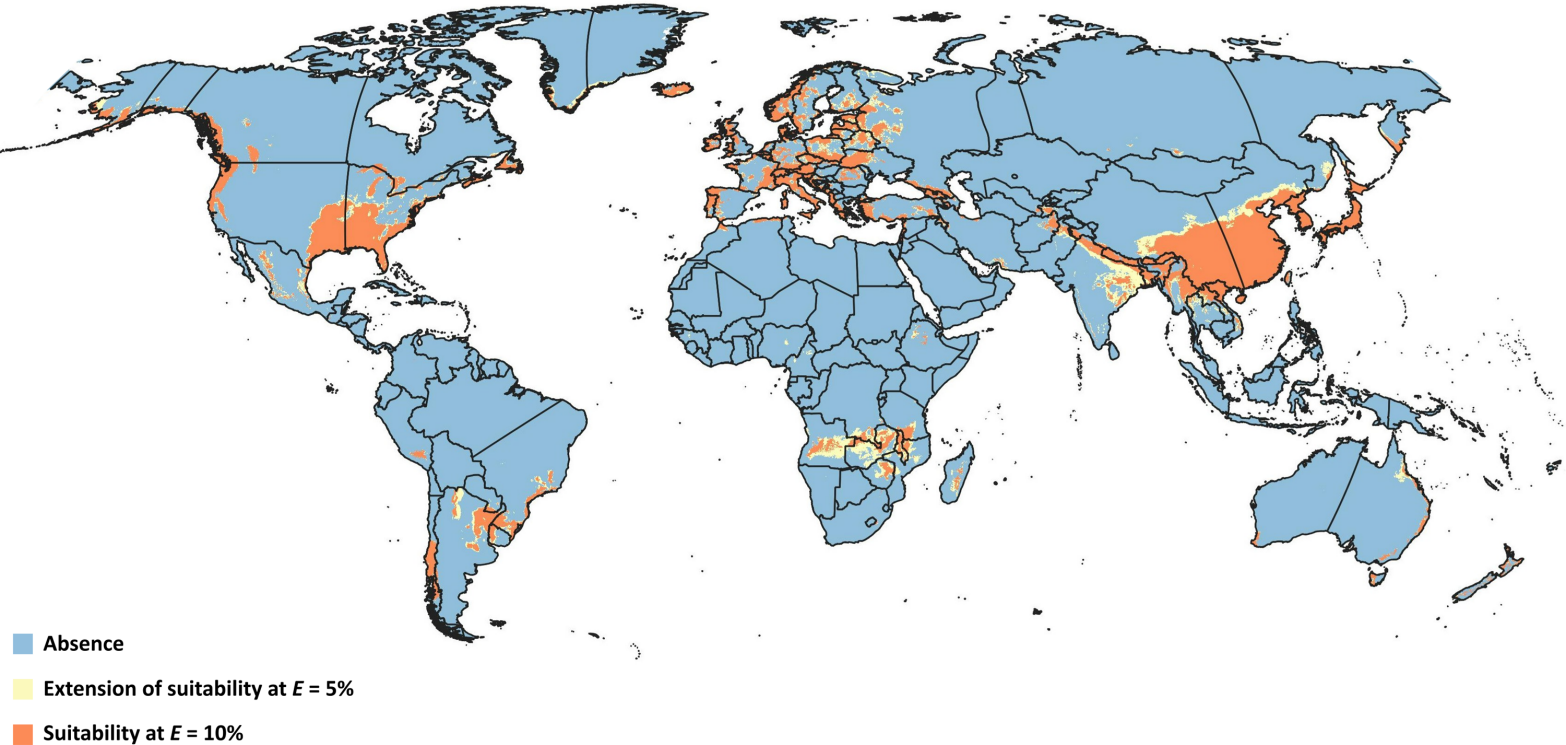
Overlapped niches. Representation of modeled suitable biocontrol areas in terms of overlapping climatic niches of *Drosophila suzukii* and *Leptopilina japonica*.

Overlapping suitable areas of *D. suzukii* and *L. japonica* to identify possible biocontrol regions for both thresholds (Fig. 4) showed that potential for *L. japonica-* mediated biocontrol management of *D. suzukii* existed in 125 nations at *E* = 5%, and 121 nations at *E* = 10% (Table 3). At a global level, the total possible biocontrol area was estimated to range 1.44 × 10^7^ km^2^−1.87 × 10^7^ km^2^ based on the different thresholds. Country-wise biocontrol coverage estimation revealed that about half of the countries (65) had more than 50% biocontrol potential (*i.e*., area suitable for both fly and wasp; Table 3), with broadest areas in China (∼4.4 × 10^6^ km^2^), India (∼1.1 × 10^6^ km^2^), Zambia (4.5 × 10^5^ km^2^), and Angola (∼4.2 × 10^5^ km^2^).

**Table 3:** Biocontrol coverage. Modeled potential for biocontrol coverage corresponding to the potential distribution of pest (*Drosophila suzukii*) and parasitoid (*Leptopilina japonica*).

**Country**	**Pest distribution (km^2^)**		**Overlapping wasp distribution (km^2^)**		**Biocontrol coverage (%)**	
	***E*= 5%**	***E*= 10%**	***E*= 5%**	***E*= 10%**	***E*= 5%**	***E*= 10%**
Afghanistan	120756.19	77038.50	87583.33	69587.73	72.53	90.33
Albania	28019.74	28019.74	27942.43	27810.06	99.72	99.25
Algeria	444458.49	222597.11	46214.07	43026.86	10.40	19.33
Andorra	452.25	452.25	407.86	344.49	90.19	76.17
Angola	725060.74	209636.29	422423.06	152662.75	58.26	72.82
Argentina	2610531.21	2136682.12	487246.09	326141.97	18.66	15.26
Armenia	29588.31	27565.71	9573.41	7333.56	32.36	26.60
Australia	4132533.88	2994970.31	284270.56	200146.28	6.88	6.68
Austria	83993.20	83993.20	77002.77	73155.41	91.68	87.10
Azerbaijan	85470.21	82660.14	29145.06	24386.68	34.10	29.50
Bahamas	9429.07	9429.07	7814.62	7766.95	82.88	82.37
Bangladesh	128942.02	101548.17	88769.51	75811.84	68.84	74.66
Belarus	207499.14	207499.14	131398.08	72083.15	63.32	34.74
Belgium	30597.07	30597.07	27115.20	24893.18	88.62	81.36
Bhutan	38954.11	37112.34	33859.02	32179.91	86.92	86.71
Bolivia	475959.05	203932.55	3394.30	1816.23	0.71	0.89
Bosnia and Herzegovina	51824.53	51824.53	32450.62	28581.47	62.62	55.15
Brazil	2088214.01	1385571.53	365390.65	278811.34	17.50	20.12
Brazilian Island	2.82	2.82	2.82	2.82	100.00	100.00
Bulgaria	112513.51	112513.51	1544.03	971.72	1.37	0.86
Cabo Verde	1750.55	630.75	479.08	269.84	27.37	42.78
Cambodia	6253.09	790.65	2055.16	133.61	32.87	16.90
Cameroon	26383.63	1809.00	14635.67	169.68	55.47	9.38
Canada	2793734.16	2155273.21	565894.51	487960.16	20.26	22.64
Chile	582097.31	475909.40	205933.41	193145.19	35.38	40.58
China	4488161.45	3862635.87	4374430.56	3827973.12	97.47	99.10
Colombia	91947.38	68214.15	340.13	340.13	0.37	0.50
Croatia	52932.84	52932.84	40105.39	36135.71	75.77	68.27
Cuba	81360.79	7022.89	510.20	26.30	0.63	0.37
Cyprus	5122.47	3433.88	4427.71	3433.88	86.44	100.00
Cyprus No Mans	296.73	33.20	72.49	33.19	24.43	99.98
Czechia	78758.87	78758.87	68665.32	58491.87	87.18	74.27
Democratic Republic of the Congo	420374.68	150483.68	161159.34	75871.68	38.34	50.42
Denmark	202079.28	130500.31	166506.11	111597.35	82.40	85.52
Djibouti	13137.52	–	467.25	–	3.56	–
Egypt	180965.62	–	120.06	–	0.07	–
Equatorial Guinea	14.17	14.17	14.17	14.17	100.00	100.00
Estonia	44389.34	44389.34	43945.97	37804.22	99.00	85.17
Ethiopia	413796.88	155614.76	39007.25	21656.50	9.43	13.92
Finland	300806.37	238124.40	93531.28	50354.32	31.09	21.15
France	562246.74	556671.13	274309.40	231951.91	48.79	41.67
Gabon	45201.71	2552.27	1222.11	267.10	2.70	10.47
Georgia	69301.13	69301.13	61604.07	58319.71	88.89	84.15
Germany	355684.24	355684.24	192739.07	159474.97	54.19	44.84
Greece	123576.16	123576.16	84346.67	79969.84	68.25	64.71
Guatemala	22913.49	16722.41	2064.41	1734.17	9.01	10.37
Guinea	4192.93	83.65	3857.20	83.65	91.99	100.00
Hungary	93200.95	93200.95	7046.93	3609.03	7.56	3.87
Iceland	98272.76	94978.48	88865.07	83482.50	90.43	87.90
India	1274811.80	685789.21	1102039.55	543419.47	86.45	79.24
Iran	336850.53	116130.87	63329.32	37732.16	18.80	32.49
Iraq	34709.55	21099.93	29371.04	19865.20	84.62	94.15
Ireland	66629.91	66332.38	55507.01	52328.26	83.31	78.89
Israel	13449.48	8745.65	11881.97	8745.68	88.35	100.00
Italy	295635.80	295613.28	264427.13	250415.73	89.44	84.71
Japan	357893.96	356945.12	357893.91	356945.07	100.00	100.00
Jordan	4921.88	507.56	1872.74	507.56	38.05	100.00
Kazakhstan	201132.14	41577.90	706.08	190.06	0.35	0.46
Kosovo	10913.08	10913.08	1765.30	1373.59	16.18	12.59
Kyrgyzstan	90256.07	43305.48	1333.13	63.53	1.48	0.15
Laos	186149.10	124451.87	149422.53	103577.26	80.27	83.23
Latvia	64162.08	64162.08	60919.43	54215.14	94.95	84.50
Lebanon	9800.04	8682.25	9001.50	8503.74	91.85	97.94
Lesotho	30106.52	30106.52	318.96	28.00	1.06	0.09
Libya	184279.76	37328.30	5699.73	4033.72	3.09	10.81
Liechtenstein	137.25	137.25	137.25	137.25	100.00	100.00
Lithuania	64816.37	64816.37	40725.99	27301.77	62.83	42.12
Luxembourg	2608.47	2608.47	2608.47	2328.89	100.00	89.28
Madagascar	187519.17	104765.96	79329.34	39438.11	42.30	37.64
Malawi	111209.70	74340.02	108138.07	72489.54	97.24	97.51
Malta	270.90	270.90	270.90	270.90	100.00	100.00
Mauritius	1802.79	94.64	1752.24	44.08	97.20	46.58
Mexico	691072.40	296644.27	251952.23	125678.99	36.46	42.37
Moldova	33206.48	33206.48	4811.96	1963.38	14.49	5.91
Monaco	3.96	3.96	3.97	3.97	100.00	100.00
Montenegro	13631.45	13631.45	11836.29	11080.44	86.83	81.29
Morocco	354222.81	165793.77	27266.40	23178.86	7.70	13.98
Mozambique	278561.34	73923.95	154695.03	54111.84	55.53	73.20
Myanmar	445821.06	375179.90	361982.68	301972.44	81.19	80.49
Namibia	66288.68	–	483.94	–	0.73	–
Nepal	145624.62	141915.07	121958.80	119500.60	83.75	84.21
Netherlands	36761.20	36260.59	17922.53	15136.66	48.75	41.74
New Zealand	217910.77	212122.44	96771.30	83214.96	44.41	39.23
Nigeria	15934.24	566.67	12716.29	337.88	79.80	59.63
North Korea	120894.87	118933.68	120894.94	118933.71	100.00	100.00
North Macedonia	25385.27	25385.27	3664.82	3358.17	14.44	13.23
Northern Cyprus	2290.99	429.38	1414.83	429.39	61.76	100.00
Norway	285817.32	263207.96	225627.24	206147.09	78.94	78.32
Oman	28552.55	1859.00	3159.53	1543.99	11.07	83.06
Pakistan	153944.54	83124.10	85069.07	49372.57	55.26	59.40
Paraguay	341582.31	230965.19	37691.58	29169.69	11.03	12.63
Peru	495995.98	431000.71	49307.80	45336.48	9.94	10.52
Poland	312841.88	312841.88	214155.94	156484.69	68.46	50.02
Portugal	89463.23	89463.23	73702.57	68237.60	82.38	76.27
Republic of Serbia	77628.71	77628.71	8713.93	5548.68	11.23	7.15
Republic of the Congo	72181.30	6283.59	446.79	26.96	0.62	0.43
Romania	235895.13	235895.13	79961.95	62526.40	33.90	26.51
Russia	3710679.26	2033909.21	662712.71	395046.93	17.86	19.42
San Marino	60.32	60.32	60.32	60.32	100.00	100.00
Slovakia	48457.79	48457.79	25232.78	22596.49	52.07	46.63
Slovenia	20295.63	20295.63	19931.57	19671.10	98.21	96.92
South Africa	902797.42	591473.81	7170.51	5131.33	0.79	0.87
South Korea	94652.90	94652.90	94652.97	94652.97	100.00	100.00
Spain	502618.58	495648.00	136222.35	112053.05	27.10	22.61
Sudan	2656.20	–	83.33	–	3.14	–
Sweden	436539.07	378204.51	158054.75	115334.67	36.21	30.50
Switzerland	41344.82	40890.87	37361.01	36701.13	90.36	89.75
Syria	22207.60	12452.65	14768.48	12044.15	66.50	96.72
Taiwan	24849.72	20605.88	21861.78	18151.55	87.98	88.09
Tajikistan	73944.07	45547.87	38519.69	25750.20	52.09	56.53
Thailand	117459.90	22050.79	103622.02	22024.89	88.22	99.88
Tunisia	78947.05	53298.18	10701.95	8871.64	13.56	16.65
Turkey	761388.06	678724.38	257025.95	224311.08	33.76	33.05
Turkmenistan	59106.76	4758.25	160.83	160.83	0.27	3.38
Ukraine	570440.95	570440.95	182074.04	152631.46	31.92	26.76
United Arab Emirates	428.91	15.84	155.89	15.84	36.35	100.00
United Kingdom	247439.54	243271.25	134076.15	124951.47	54.19	51.36
Tanzania	540638.96	197481.86	110629.43	79486.65	20.46	40.25
United States of America	7267495.86	5752263.93	2879003.63	2546609.52	39.61	44.27
Uruguay	176465.55	176465.55	75249.12	56325.26	42.64	31.92
Uzbekistan	64090.46	22578.88	12752.88	9003.24	19.90	39.87
Vatican	0.01	0.01	0.01	0.01	100.00	100.00
Vietnam	208079.26	173719.30	179340.06	158519.90	86.19	91.25
Zambia	471456.61	148179.91	452563.86	146359.35	95.99	98.77
Zimbabwe	186694.49	78523.17	135058.40	69964.04	72.34	89.10

**Notes.**

E indicates thresholding level.

## Discussion

Extremely fast range expansion as a consequence of globalization (Iacovone et al., 2015), with severe economic damage to the fruit trade industry (Bolda, Goodhue & Zalom, 2010; Gabarra et al., 2015), has led to efforts to model ecological niches and predict potential distributions for *D. suzukii* both locally (Castro-Sosa et al., 2017; De la Vega & Corley, 2019) and globally (Santos et al., 2017; Ørsted & Ørsted, 2019; Reyes & Lira-Noriega, 2020). Comparing with previous global-scale models, our models predicted highly suitable areas for *D. suzukii* most similar to the model developed by Ørsted & Ørsted (2019), and less similar to those of Santos et al. (2017) and Reyes & Lira-Noriega (2020). Relatively broad geographic areas in the southern part of central and eastern Africa were predicted to be suitable in the models developed by Santos et al. (2017) and Reyes & Lira-Noriega (2020) compared to our model and that of Ørsted & Ørsted (2019). Unlike the predictions of Santos et al. (2017) and Reyes & Lira-Noriega (2020), Patagonian region of Argentina was not included as suitable habitat for *D. suzukii* in our model and that of Ørsted & Ørsted (2019). Another major difference between our model and those of Santos et al. (2017) and Reyes & Lira-Noriega (2020) is that their models predicted a large extent of eastern India as suitable habitats for *D. suzukii*. However, according to our model, the suitability was more prominent in far north, and also in some parts of Western Ghats in southern India. Although similar in many aspects of predicted distributions, our model differed notably from that of Ørsted & Ørsted (2019) in predicting the east–west continuity of potential distribution of *D. suzukii* in United States as our model showed a discontinuous distribution of potential habitats.

In exploring bioclimatic variable contributions to the pest model, mean temperature of coldest quarter and minimum temperature of coldest month both had contributions to the models (Table 2) that were substantive enough to merit comment; similar observations of the influence of cold temperatures on *D. suzukii* distribution were made by Ørsted & Ørsted (2019). Limiting influence of winter temperatures on the establishment of *D. suzukii* populations is evident from the facts that prolonged low temperature exposure (<10°) is detrimental for its viability (Dalton et al., 2011). A recent meta-analysis (Ørsted et al., 2021) revealed that temperature extremes are highly significant in determining the survival and population activity of the species. Preference of *D. suzukii* for humid environments (Gutierrez, Ponti & Dalton, 2016; Santos et al., 2017; Ørsted & Ørsted, 2019) was reflected in our models *via* high contributions of precipitation of driest quarter and precipitation of driest month. Large contributions of isothermality, precipitation seasonality, precipitation of wettest month, precipitation of driest month, and precipitation of wettest quarter to *L. japonica* model (Table 2) indicate that temperature fluctuations and humidity of environments may also play crucial roles in constraining the distribution of *L. japonica*.

For obvious reasons, choosing biological control agents for *D. suzukii* that have niche preferences similar to those of the fly will be helpful (Robertson, Kriticos & Zachariades, 2008; Olfert et al., 2016; Tepa-Yotto et al., 2021a; Tepa-Yotto et al., 2021b) in the global-scale biological control challenge. Matching the climatic niche requirements of pest and parasitoid will increase chances of long-term establishment of the parasitoid across key regions (Robertson, Kriticos & Zachariades, 2008), resulting in more successful management *via* biological control. Despite various previous studies modeling the climatic niche of *D. suzukii*, to the best our knowledge, no effort has been made so far to study the potential distribution of climatic niches of any parasitoid of *D. suzukii* in combination with analyses of the climatic niche of the fly pest.

Range expansion of *D. suzukii* in Europe and North America occurred after initial outbreaks in California, Spain, and Italy, all in 2008 (Rota-Stabelli, Blaxter & Anfora, 2013; Asplen et al., 2015). Niche filling related to absence of competitors or natural enemies, high adaptability to temperate climates, high dispersal ability, and high reproductive output, are major factors contributing to the unprecedented invasion of *D. suzukii* (Rota-Stabelli, Blaxter & Anfora, 2013). As niche filling is an important factor, assessing the geographic distribution of climatic niches of *D. suzukii* becomes an indispensable step in biological control programs, as it can provide an initial estimate of the geographic limits for successful parasitoid release (Puppato et al., 2020). Development of niche models for parasitoids, and identification of geographic regions exhibiting overlapping climatic niches between pest and parasitoid, further delimits regions for parasitoid release, making field trials involving elaborate and time-consuming experiments more economical (Sun et al., 2017).

In its native distributional areas, *L. japonica* is one of most abundant potential parasitoids of *D. suzukii* (Kimura & Novković, 2015; Puppato et al., 2020); its occurrence in Europe(Puppato et al., 2020) and North America (Abram et al., 2020; Abram et al., 2022; Beers et al., 2022) was identified only recently. Previous laboratory experiments in the United States indicated that South Korean *L. japonica* strains attacked the North American strains of *D. suzukii* readily (Daane et al., 2016), supporting at least in part the suitability of *L. japonica* as a biocontrol agent for *D. suzukii*. Although occurrence records of *L. japonica* were scarce, our modeled climatic niche for *L. japonica* overlapped broadly with that of *D. suzukii* in known-invaded regions (Figs. 2–4), meeting one of the major ecological requirements for a ‘natural enemy species’ to be a candidate biological control agent (Robertson, Kriticos & Zachariades, 2008; Olfert et al., 2016).

In addition, our statistical quantification of similarity or difference in ecological requirements of pest and parasitoid failed to reject the null hypothesis of niche similarity (Peterson, 2011; Tocchio et al., 2015), revealing that their overlapping niches are similar, at least given the data available to us. Using overlap of potential distributions in geographic space, Tepa-Yotto et al. (2021b) explored the possibility of ENM in devising biological control measures for the fall armyworm, *Spodoptera frugiperda*, as regards its key parasitoids. In terms of the Hutchinsonian duality, overlapping potential distributions of pest and parasitoid in geographic space alone does not help researchers to conclude that ecological requirements of the species are same because a point in geographic space can be expressed as only one point in environmental space but a point in environmental space may expressed by more than one point in geographic space (Castaneda-Guzman, 2022; Nuñez Penichet et al., 2022). Considering niche overlap of pest and parasitoid in both geographic space and environmental space is therefore essential to confirm that the species can indeed interact. Ability of the biocontrol agent to colonize the full distributional area of the target species is critical for the success of biocontrol programs (Gupta et al., 2022). The two-species model developed for gaining insight into the biotic interactions shaping the potential geographic distribution of *L. japonica* underperformed compared to the climate-only model. These results thus contrasted with previous findings highlighting the importance of including biotic predictors in ecological niche modeling procedures to improve model performance (Araújo & Luoto, 2007; Giannini et al., 2013; Dormann et al., 2018; Simões & Peterson, 2018; Bebber & Gurr, 2019; Ashraf, Chaudhry & Peterson, 2021).

We recommend a niche-based, target-oriented prioritization approach in designing biological control programs aimed at *D. suzukii.* In Europe, three interlinked factors, (1) recently recorded occurrences (Puppato et al., 2020), (2) predicted suitability in 17 European countries (∼39% of European countries) with biocontrol coverage of >80% at both thresholds (*E* = 5% and *E* = 10%)(Table 3), and (3) increasing consumer preference towards organic fruits (Murphy et al., 2022), make *L. japonica* a candidate parasitoid for control of *D. suzukii*. In the remaining European countries, in particular those exhibiting biocontrol coverage <50%, we suggest extra care in defining appropriate geographic boundaries for *L. japonica* release plans (Table 3). In the United States and Canada, the potential distribution of *L. japonica* overlapped only one-third of *D. suzukii’s* potential distributional area, demanding strict site-specific release planning. Site-specific pest management utilizing pest distributional information is preferred over uniform pest management (Park, Krell & Carroll, 2007). However, for effective site-specific biological control of pests, not only the pest distributional information but also the niche overlap between pest and parasitoids must be taken into account. Irrespective of the biocontrol coverage in *D. suzukii* invaded regions, any *L. japonica* release strategy has to rely not only on specific details of both site and niche considerations but on host-specificity trials by taking non-target insects from the local fauna also into account (Van Driesche & Hoddle, 1997).

In a study that tested the host specificity of three parasitoids of *D. suzukii* (Daane et al., 2021), highly host-specific *G. brasiliensis* (Wang et al., 2020; Daane et al., 2021) successfully parasitized the target *D. suzukii* and three other related species (*D. simulans, D. melanogaster*, and *D. persimilis*). Our species of interest (*L. japonica*) was successful in parasitizing *D. suzukii* at a high rate, as well as nine other related species (*D. simulans, D. melanogaster*, *D. persimilis, D. montana*, *D. robusta*, *D. tripunctata*, *D. willistoni*, *D. funebris*, *Hirtodrosophila duncani*) at relatively lower rates. Taking these facts into consideration, prior to development of *L. japonica* release plans, care must be taken to properly address two important questions: what is expected range of hosts to be parasitized, and what are the ecological and economic values that we place on them (Van Driesche & Hoddle, 1997), as well as real-world performance of the parasitoid in small-scale release experiments.

Gross national income (GNI) per capita of the exporting country and incidences of interception of insect pests at international ports of entry are known to be negatively correlated (Liebhold et al., 2006), such that lower-income countries are at a greater risk of pest-induced crop damage (Gaiha et al., 2009) due to poor surveillance (Liebhold et al., 2006). More than 80% of current low-income economies are in Africa (https://datahelpdesk.worldbank.org, accessed on 21/01/2023); based on the biocontrol coverage explored in this paper, the possibility of applying *L. japonica* mediated biocontrol measures exists in six low-income African countries (Republic of the Congo, Guinea, Madagascar, Malawi, Mozambique, Zambia). Considering the low GNI per capita, and significant export potential of fruits from Malawi (tropical fruits; US$1.41M/year) and Mozambique (tropical fruits; US$3.77M/year) (The observatory of economic complexity data, https://oec.world, accessed on 21/01/2023), *D. suzukii* invasion in these sub-Saharan African (SSA) countries can affect not only the fruit yield within these countries but also fruit cultivation of the countries of import. Biocontrol measures reduce insect pest multiplication and benefit crop yields in SSA, and large-scale biocontrol programs can enhance food security in this region (Ratto et al., 2022). We recommended extensive field surveys in Malawi and Mozambique to check for presence of *D. suzukii*; if presence is confirmed, adopting biocontrol strategies in the national and regional farming policies of these countries may produce the double benefit of increasing crop yield within the countries, and reducing possible transnational crossing of *D. suzukii* to the countries of import. Biological control in SSA comes under the purview of the consultative group on international agricultural research (CGIAR) (Adenle, Wedig & Azadi, 2019). Given that the presence of *D. suzukii* has already been confirmed in SSA (Kwadha et al., 2021) and that domestic research facilities are relatively poor (Pal, 2011), intervention of multilateral development agencies like CGIAR in detecting invaded insect pests in agricultural fields, estimating its abundance, and devising effective strategies of biological control is recommended (Adenle, Wedig & Azadi, 2019).

In conclusion, this study illustrates a cost-effective pre-assessment strategy that can be applied to any biological control management program before beginning the labor-intensive, time-consuming, and expensive field experiments. Availability of a greater number of occurrence records of *L. japonica* would further enhance the understanding of the distributional potential of this potential biocontrol agent worldwide. Of course, we do not recommend straight-away release of *L. japonica* into the fields where biocontrol of *D. suzukii* may be potentially beneficial to the farming community. We suggest instead to treat this study as a preliminary platform in general to develop a niche-based, target-oriented prioritization approach to select potential species for biocontrol management with the support of evidences from host range trials involving choice and no-choice considerations.

##  Supplemental Information

10.7717/peerj.15222/supp-1 File S1 Occurrence records of invasive pest *D. suzukii* Presence points of *D. suzukii* used for global-scale ecological niche modeling

10.7717/peerj.15222/supp-2 File S2 Occurrence records of parasitoid *L. japonica* Presence points of parasitoid *L. japonica* used for global-scale ecological niche modeling

10.7717/peerj.15222/supp-3 File S3 Estimation of country-wise area of potential habitats Country-wise area estimation of potential habitats for both pest and parasitoid at most desirable (E = 5%) and maximum permitted (E = 10%) thresholds.

10.7717/peerj.15222/supp-4 File S4 Niche similarity testing Niches of pest and parasitoid are represented along the first two principal components (top panel). Dark shading indicates the occurrence densities of pest and parasitoid by cell. The solid and dashed contour lines indicate 100% and 50% of background environment in the area **M**. The empirical D value fell within the null distribution of D values generated in both tests (Broennimann et al., 2012, bottom left; Warren, Glor & Turelli, 2008, bottom right), indicating the non-rejection of the null hypothesis of niche similarity. E-space: Environmental space, G-space: Geographic space

## Additional Information and Declarations

### Data Availability

The following information was supplied regarding data availability: The presence points used for modeling potential habitats of pest and parasitoid, and the country-wise area calculations associated with different thresholds (5% and 10%) for both pest and parasitoid are available in the Supplementary Files.

## References

[ref-1] Abram PK, Franklin MT, Hueppelsheuser T, Carrillo J, Grove E, Eraso P, Acheampong S, Keery L, Girod P, Tsuruda M, Clausen M, Buffington ML, Moffat CE (2022). Adventive larval parasitoids reconstruct their close association with spotted-wing *Drosophila* in the invaded North American range. Environmental Entomology.

[ref-2] Abram PK, McPherson AE, Kula R, Hueppelsheuser T, Thiessen J, Perlman SJ, Curtis CI, Fraser JL, Tam J, Carrillo J, Gates M, Scheffer S, Lewis M, Buffington M (2020). New records of *Leptopilina*, Ganaspis, and *Asobara* species associated with *Drosophila suzukii* in North America, including detections of *L. japonica* and *G. brasiliensis*. Journal of Hymenoptera Research.

[ref-3] Adenle AA, Wedig K, Azadi H (2019). Sustainable agriculture and food security in Africa: the role of innovative technologies and international organizations. Technology in Society.

[ref-4] Agboka KM, Tonnang HEZ, Abdel-Rahman EM, Kimathi E, Mutanga O, Odindi J, Niassy S, Mohamed SA, Ekesi S (2022). A systematic methodological approach to estimate the impacts of a classical biological control agent’s dispersal at landscape: application to fruit fly *Bactrocera dorsalis* and its endoparasitoid *Fopius arisanus*. Biological Control.

[ref-5] Aiello-Lammens ME, Boria RA, Radosavljevic A, Vilela B, Anderson RP (2015). spThin: an R package for spatial thinning of species occurrence records for use in ecological niche models. Ecography.

[ref-6] Andreazza F, Bernardi D, Dos Santos RSS, Garcia FRM, Oliveira EE, Botton M, Nava DE (2017). Drosophila suzukii in southern Neotropical region: current status and future perspectives. Neotropical Entomology.

[ref-7] Araújo MB, Luoto M (2007). The importance of biotic interactions for modelling species distributions under climate change. Global Ecology and Biogeography.

[ref-8] Ashraf U, Chaudhry MN, Peterson AT (2021). Ecological niche models of biotic interactions predict increasing pest risk to olive cultivars with changing climate. Ecosphere.

[ref-9] Asplen MK, Anfora G, Biondi A, Choi D-S, Chu D, Daane KM, Gibert P, Gutierrez AP, Hoelmer KA, Hutchison WD, Isaacs R, Jiang Z-L, Kárpáti Z, Kimura MT, Pascual M, Philips CR, Plantamp C, Ponti L, Vétek G, Vogt H, Walton VM, Yu Y, Zappalà L, Desneux N (2015). Invasion biology of spotted wing *Drosophila* (*Drosophila suzukii*): a global perspective and future priorities. Journal of Pest Science.

[ref-10] Barrows CW, Preston KL, Rotenberry JT, Allen MF (2008). Using occurrence records to model historic distributions and estimate habitat losses for two psammophilic lizards. Biological Conservation.

[ref-11] Barve N, Barve V, Jiménez-Valverde A, Lira-Noriega A, Maher SP, Peterson AT, Soberón J, Villalobos F (2011). The crucial role of the accessible area in ecological niche modeling and species distribution modeling. Ecological Modelling.

[ref-12] Bebber DP, Gurr SJ (2019). Biotic interactions and climate in species distribution modelling.

[ref-13] Beers EH, Beal D, Smytheman P, Abram PK, Schmidt-Jeffris R, Moretti E, Daane KM, Looney C, Lue C-H, Buffington M (2022). First records of adventive populations of the parasitoids *Ganaspis brasiliensis* and *Leptopilina japonica* in the United States. Journal of Hymenoptera Research.

[ref-14] Beers EH, Van Steenwyk RA, Shearer PW, Coates WW, Grant JA (2011). Developing *Drosophila suzukii* management programs for sweet cherry in the western United States. Pest Management Science.

[ref-15] Bellamy DE, Sisterson MS, Walse SS (2013). Quantifying host potentials: indexing postharvest fresh fruits for spotted wing *Drosophila*, Drosophila suzukii. PLOS ONE.

[ref-16] Benito NP, Lopes-da Silva M, Santos RSS dos (2016). Potential spread and economic impact of invasive *Drosophila suzukii* in Brazil. Pesquisa Agropecuária Brasileira.

[ref-17] Bernaola L, Holt JR (2021). Incorporating sustainable and technological approaches in pest management of invasive arthropod species. Annals of the Entomological Society of America.

[ref-18] Biondi A, Wang X, Daane KM (2021). Host preference of three Asian larval parasitoids to closely related *Drosophila* species: implications for biological control of *Drosophila suzukii*. Journal of Pest Science.

[ref-19] Bogosian III V, Hellgren EC, Sears MW, Moody RW (2012). High-resolution niche models *via* a correlative approach: comparing and combining correlative and process-based information. Ecological Modelling.

[ref-20] Bolda MP, Goodhue RE, Zalom FG (2010). Spotted wing *Drosophila*: potential economic impact of a newly established pest. Agricultural and Resource Economics Update.

[ref-21] Booth TH (2022). Checking bioclimatic variables that combine temperature and precipitation data before their use in species distribution models. Austral Ecology.

[ref-22] Bramlett M, Plaetinck G, Maienfisch P (2020). RNA-based biocontrols—a new paradigm in crop protection. Engineering.

[ref-23] Broennimann O, Fitzpatrick MC, Pearman PB, Petitpierre B, Pellissier L, Yoccoz NG, Thuiller W, Fortin M-J, Randin C, Zimmermann NE, Graham CH, Guisan A (2012). Measuring ecological niche overlap from occurrence and spatial environmental data. Global Ecology and Biogeography.

[ref-24] Calabria G, Máca J, Bächli G, Serra L, Pascual M (2012). First records of the potential pest species *Drosophila suzukii* (Diptera: Drosophilidae) in Europe. Journal of Applied Entomology.

[ref-25] Castaneda-Guzman M (2022). Modeling species geographic distributions in aquatic ecosystems using a density-based clustering algorithm.

[ref-26] Castro-Sosa R, Castillo-Peralta M del R, Monterroso-Rivas AI, Gomez-Díaz JD, Flores-González E, Rebollar-Alviter Á (2017). Potential distribution of *Drosophila suzukii* (Diptera: Drosophilidae) in relation to alternate hosts in Mexico. Florida Entomologist.

[ref-27] Chabert S, Allemand R, Poyet M, Eslin P, Gibert P (2012). Ability of European parasitoids (Hymenoptera) to control a new invasive Asiatic pest, *Drosophila suzukii*. Biological Control.

[ref-28] Chamberlain S (2016). sCrubr: Clean biological occurrence records (see https://rdrr.io/github/ropensci/scrubr/). https://rdrr.io/github/ropensci/scrubr/.

[ref-29] Chamberlain S, Ram K, Hart T (2021). spocc: interface to species occurrence data sources (see http://CRAN.R-project.org/package=spocc). R package version 1.2.0. http://CRAN.R-project.org/packagespocc.

[ref-30] Cloonan KR, Abraham J, Angeli S, Syed Z, Rodriguez-Saona C (2018). Advances in the chemical ecology of the spotted wing *Drosophila* (*Drosophila suzukii*) and its applications. Journal of Chemical Ecology.

[ref-31] Cobos ME, Peterson AT, Barve N, Osorio-Olvera L (2019). Kuenm: an R package for detailed development of ecological niche models using Maxent. PeerJ.

[ref-32] Cock MJW, Lenteren JC Van, Brodeur J, Barratt BIP, Bigler F, Bolckmans K, Cônsoli FL, Haas F, Mason PG, Parra JRP (2010). Do new access and benefit sharing procedures under the convention on biological diversity threaten the future of biological control?. BioControl.

[ref-33] Cruz-Cárdenas G, López-Mata L, Villaseñor JL, Ortiz E (2014). Potential species distribution modeling and the use of principal component analysis as predictor variables. Revista Mexicana de Biodiversidad.

[ref-34] Daane KM, Wang X-G, Biondi A, Miller B, Miller JC, Riedl H, Shearer PW, Guerrieri E, Giorgini M, Buffington M, Van Achterberg K, Song Y, Kang T, Yi H, Jung C, Lee DW, Chung B-K, Hoelmer KA, Walton VM (2016). First exploration of parasitoids of *Drosophila suzukii* in South Korea as potential classical biological agents. Journal of Pest Science.

[ref-35] Daane KM, Wang X, Hogg BN, Biondi A (2021). Potential host ranges of three Asian larval parasitoids of *Drosophila suzukii*. Journal of Pest Science.

[ref-36] Dalton DT, Walton VM, Shearer PW, Walsh DB, Caprile J, Isaacs R (2011). Laboratory survival of *Drosophila suzukii* under simulated winter conditions of the Pacific Northwest and seasonal field trapping in five primary regions of small and stone fruit production in the United States. Pest Management Science.

[ref-37] Dara SK, Awasthi LP (2021). Integrated insect pest management of economically important crops. Biopesticides in organic farming.

[ref-38] De la Vega GJ, Corley JC (2019). *Drosophila suzukii* (Diptera: Drosophilidae) distribution modelling improves our understanding of pest range limits. International Journal of Pest Management.

[ref-39] Demján P, Dreslerová D, Kolář J, Chuman T, Romportl D, Trnka M, Lieskovský T (2022). Long time-series ecological niche modelling using archaeological settlement data: tracing the origins of present-day landscape. Applied Geography.

[ref-40] Deprá M, Poppe JL, Schmitz HJ, De Toni DC, Valente VLS (2014). The first records of the invasive pest *Drosophila suzukii* in the South American continent. Journal of Pest Science.

[ref-41] Di Cola V, Broennimann O, Petitpierre B, Breiner FT, D’Amen M, Randin C, Engler R, Pottier J, Pio D, Dubuis A, Pellissier L, Mateo RG, Hordijk W, Salamin N, Guisan A (2017). ecospat: an R package to support spatial analyses and modeling of species niches and distributions. Ecography.

[ref-42] DiGiacomo G, Hadrich J, Hutchison WD, Peterson H, Rogers M (2019). Economic impact of spotted wing *Drosophila* (Diptera: Drosophilidae) yield loss on Minnesota raspberry farms: a grower survey. Journal of Integrated Pest Management.

[ref-43] Dormann CF, Bobrowski M, Dehling DM, Harris DJ, Hartig F, Lischke H, Moretti MD, Pagel J, Pinkert S, Schleuning M, Schmidt SI, Sheppard CS, Steinbauer MJ, Zeuss D, Kraan C (2018). Biotic interactions in species distribution modelling: 10 questions to guide interpretation and avoid false conclusions. Global Ecology and Biogeography.

[ref-44] Escobar LE (2020). Ecological niche modeling: an introduction for veterinarians and epidemiologists. Frontiers in Veterinary Science.

[ref-45] Escobar LE, Lira-Noriega A, Medina-Vogel G, Peterson AT (2014). Potential for spread of the white-nose fungus (*Pseudogymnoascus destructans*) in the Americas: use of Maxent and NicheA to assure strict model transference. Geospatial Health.

[ref-46] Fanning PD, Grieshop MJ, Isaacs R (2018). Efficacy of biopesticides on spotted wing *Drosophila*, Drosophila suzukii Matsumura in fall red raspberries. Journal of Applied Entomology.

[ref-47] Farnsworth D, Hamby KA, Bolda M, Goodhue RE, Williams JC, Zalom FG (2017). Economic analysis of revenue losses and control costs associated with the spotted wing *Drosophila*, Drosophila suzukii (Matsumura), in the California raspberry industry. Pest Management Science.

[ref-48] Fick SE, Hijmans RJ (2017). WorldClim 2: new 1-km spatial resolution climate surfaces for global land areas. International Journal of Climatology.

[ref-49] Food and Agriculture Organization of the United Nations (ed.) (2013). The state of food insecurity in the world 2013: the multiple dimensions of food security.

[ref-50] Furihata S, Matsumura T, Hirata M, Mizutani T, Nagata N, Kataoka M, Katayama Y, Omatsu T, Matsumoto H, Hayakawa Y (2016). Characterization of venom and oviduct components of parasitoid wasp *Asobara japonica*. PLOS ONE.

[ref-51] Gabarra R, Riudavets J, Rodríguez GA, Pujade-Villar J, Arnó J (2015). Prospects for the biological control of *Drosophila suzukii*. BioControl.

[ref-52] Gaiha R, Imai K, Hill K, Mathur S (2009). On insect infestation and agricultural productivity in developing countries. Economics Discussion Paper Series—0910.

[ref-53] Giannini TC, Chapman DS, Saraiva AM, Alves-dos Santos I, Biesmeijer JC (2013). Improving species distribution models using biotic interactions: a case study of parasites, pollinators and plants. Ecography.

[ref-54] Giorgini M, Wang X-G, Wang Y, Chen F-S, Hougardy E, Zhang H-M, Chen Z-Q, Chen H-Y, Liu C-X, Cascone P, Formisano G, Carvalho GA, Biondi A, Buffington M, Daane KM, Hoelmer KA, Guerrieri E (2019). Exploration for native parasitoids of *Drosophila suzukii* in China reveals a diversity of parasitoid species and narrow host range of the dominant parasitoid. Journal of Pest Science.

[ref-55] Girod P (2018). From Asia to Europe, evaluation of parasitoids for the biological control of the invasive fruit pest *Drosophila suzukii*.

[ref-56] Girod P, Lierhmann O, Urvois T, Turlings TCJ, Kenis M, Haye T (2018). Host specificity of Asian parasitoids for potential classical biological control of *Drosophila suzukii*. Journal of Pest Science.

[ref-57] Goodhue RE, Bolda M, Farnsworth D, Williams JC, Zalom FG (2011). Spotted wing *Drosophila* infestation of California strawberries and raspberries: economic analysis of potential revenue losses and control costs. Pest Management Science.

[ref-58] Gupta S, Choudhary M, Singh B, Singh R, Dhar MK, Kaul S (2022). Diversity and biological activity of fungal endophytes of Zingiber officinale Rosc. with emphasis on Aspergillus terreus as a biocontrol agent of its leaf spot. Biocatalysis and Agricultural Biotechnology.

[ref-59] Gutierrez AP, Ponti L, Dalton DT (2016). Analysis of the invasiveness of spotted wing *Drosophila* (*Drosophila suzukii*) in North America, Europe, and the Mediterranean Basin. Biological Invasions.

[ref-60] Hauser M (2011). A historic account of the invasion of *Drosophila suzukii* (Matsumura) (Diptera: Drosophilidae) in the continental United States, with remarks on their identification. Pest Management Science.

[ref-61] Haye T, Girod P, Cuthbertson AGS, Wang XG, Daane KM, Hoelmer KA, Baroffio C, Zhang JP, Desneux N (2016). Current SWD IPM tactics and their practical implementation in fruit crops across different regions around the world. Journal of Pest Science.

[ref-62] Iacovone A, Girod P, Ris N, Weydert C, Gibert P, Poirié M, Gatti J-L (2015). Worldwide invasion by *Drosophila suzukii*: does being the cousin of a model organism really help setting up biological control? hopes, disenchantments and new perspectives. Revue d’Ecologie, Terre et Vie.

[ref-63] Ideo S, Watada M, Mitsui H, Kimura MT (2008). Host range of *Asobara japonica* (Hymenoptera: Braconidae), a larval parasitoid of drosophilid flies. Entomological Science.

[ref-64] Júnior PDM, Nóbrega CC (2018). Evaluating collinearity effects on species distribution models: an approach based on virtual species simulation. PLOS ONE.

[ref-65] Kacsoh BZ, Schlenke TA (2012). High hemocyte load is associated with increased resistance against parasitoids in *Drosophila suzukii*, a relative of *D. melanogaster*. PLOS ONE.

[ref-66] Kanzawa T (1935). Research into the fruit-fly *Drosophila suzukii* Matsumura (preliminary report).

[ref-67] Kehrli P, Cruchon Y, Stäheli N, Cara C, Linder C (2017). Drosophila suzukii: un ravageur principal du vignoble?. Revue Suisse de Viticulture, Arboriculture, Horticulture.

[ref-68] Kienzle R, Rohlfs M (2021). Mind the wound!—fruit injury ranks higher than, and interacts with, heterospecific cues for *Drosophila suzukii* oviposition. Insects.

[ref-69] Kimura MT, Novković B (2015). Local adaptation and ecological fitting in host use of the *Drosophila* parasitoid *Leptopilina japonica*. Ecological Research.

[ref-70] Knapp L, Mazzi D, Finger R (2019). Management strategies against *Drosophila suzukii*: insights into Swiss grape growers choices. Pest Management Science.

[ref-71] Knapp L, Mazzi D, Finger R (2021). The economic impact of *Drosophila suzukii*: perceived costs and revenue losses of Swiss cherry, plum and grape growers. Pest Management Science.

[ref-72] Knoll V, Ellenbroek T, Romeis J, Collatz J (2017). Seasonal and regional presence of hymenopteran parasitoids of *Drosophila* in Switzerland and their ability to parasitize the invasive *Drosophila suzukii*. Scientific Reports.

[ref-73] Kolanowska M, Jakubska-Busse A (2020). Is the lady’s-slipper orchid (*Cypripedium calceolus*) likely to shortly become extinct in Europe?—insights based on ecological niche modelling. PLOS ONE.

[ref-74] Kruitwagen A, Beukeboom LW, Wertheim B (2018). Optimization of native biocontrol agents, with parasitoids of the invasive pest *Drosophila suzukii* as an example. Evolutionary Applications.

[ref-75] Kwadha CA, Okwaro LA, Kleman I, Rehermann G, Revadi S, Ndlela S, Khamis FM, Nderitu PW, Kasina M, George MK, Kithusi GG, Mohamed SA, Lattorff HMG, Becher PG (2021). Detection of the spotted wing *Drosophila*, Drosophila suzukii, in continental sub-Saharan Africa. Journal of Pest Science.

[ref-76] Landolt PJ, Adams T, Rogg H (2012). Trapping spotted wing *Drosophila*, Drosophila suzukii (Matsumura) (Diptera: Drosophilidae), with combinations of vinegar and wine, and acetic acid and ethanol. Journal of Applied Entomology.

[ref-77] Lee JC, Bruck DJ, Dreves AJ, Ioriatti C, Vogt H, Baufeld P (2011). In focus: spotted wing *Drosophila*, Drosophila suzukii, across perspectives. Pest Management Science.

[ref-78] Lee JC, Dalton DT, Swoboda-Bhattarai KA, Bruck DJ, Burrack HJ, Strik BC, Woltz JM, Walton VM (2016). Characterization and manipulation of fruit susceptibility to *Drosophila suzukii*. Journal of Pest Science.

[ref-79] Liebhold AM, Work TT, McCullough DG, Cavey JF (2006). Airline baggage as a pathway for alien insect species invading the United States. American Entomologist.

[ref-80] Louis C, Girard M, Kuhl G, Lopez-Ferber M (1996). Persistence of *Botrytis cinerea* in its vector *Drosophila melanogaster*. Phytopathology.

[ref-81] Machado-Stredel F, Cobos ME, Peterson AT (2021). A simulation-based method for selecting calibration areas for ecological niche models and species distribution models. Frontiers of Biogeography.

[ref-82] Mazzetto F, Marchetti E, Amiresmaeili N, Sacco D, Francati S, Jucker C, Dindo ML, Lupi D, Tavella L (2016). Drosophila parasitoids in northern Italy and their potential to attack the exotic pest *Drosophila suzukii*. Journal of Pest Science.

[ref-83] Mills NJ (2018). An alternative perspective for the theory of biological control. Insects.

[ref-84] Mitsui H, Takahashi KH, Kimura MT (2006). Spatial distributions and clutch sizes of *Drosophila* species ovipositing on cherry fruits of different stages. Population Ecology.

[ref-85] Molina JJ, Harrison MD, Brewer JW (1974). Transmission of *Erwinia carotovora* var. atroseptica by *Drosophila melanogaster* Meig. I. Acquisition and transmission of the bacterium. American Potato Journal.

[ref-86] Murphy B, Martini M, Fedi A, Loera BL, Elliott CT, Dean M (2022). Consumer trust in organic food and organic certifications in four European countries. Food Control.

[ref-87] Murphy KA, Tabuloc CA, Cervantes KR, Chiu JC (2016). Ingestion of genetically modified yeast symbiont reduces fitness of an insect pest *via* RNA interference. Scientific Reports.

[ref-88] Novković B, Mitsui H, Suwito A, Kimura MT (2011). Taxonomy and phylogeny of *Leptopilina* species (Hymenoptera: Cynipoidea: Figitidae) attacking frugivorous drosophilid flies in Japan, with description of three new species. Entomological Science.

[ref-89] Nuñez Penichet C, Cobos ME, Soberón J, Gueta T, Barve N, Barve V, Navarro-Sigüenza AG, Peterson AT (2022). Selection of sampling sites for biodiversity inventory: effects of environmental and geographical considerations. Methods in Ecology and Evolution.

[ref-90] Pérez-de la O NB, Espinosa-Zaragoza S, López-Martínez V, Hight DS, Varone L (2020). Ecological niche modeling to calculate ideal sites to introduce a natural enemy: the case of *Apanteles opuntiarum* (Hymenoptera: Braconidae) to control *Cactoblastis cactorum* (Lepidoptera: Pyralidae) in North America. Insects.

[ref-91] Olfert O, Haye T, Weiss R, Kriticos D, Kuhlmann U (2016). Modelling the potential impact of climate change on future spatial and temporal patterns of biological control agents: *Peristenus digoneutis* (Hymenoptera: Braconidae) as a case study. The Canadian Entomologist.

[ref-92] Ørsted M, Lye J, Umina PA, Maino JL (2021). Global analysis of the seasonal abundance of the invasive pest *Drosophila suzukii* reveal temperature extremes determine population activity potential. Pest Management Science.

[ref-93] Ørsted IV, Ørsted M (2019). Species distribution models of the spotted wing *Drosophila* (*Drosophila suzukii*, Diptera: Drosophilidae) in its native and invasive range reveal an ecological niche shift. Journal of Applied Ecology.

[ref-94] Outammassine A, Zouhair S, Loqman S (2022). Global potential distribution of three underappreciated arboviruses vectors (*Aedes japonicus*, Aedes vexans and *Aedes vittatus*) under current and future climate conditions. Transboundary and Emerging Diseases.

[ref-95] Pal S (2011). Impacts of CGIAR crop improvement and natural resource management research: a review of evidence.

[ref-96] Park Y-L, Krell RK, Carroll M (2007). Theory, technology, and practice of site-specific insect pest management. Journal of Asia-Pacific Entomology.

[ref-97] Peterson AT (2011). Ecological niche conservatism: a time-structured review of evidence. Journal of Biogeography.

[ref-98] Peterson AT (2012). Niche modeling—model evaluation. Biodiversity Informatics.

[ref-99] Peterson AT, Ball LG, Cohoon KP (2002). Predicting distributions of Mexican birds using ecological niche modelling methods. Ibis.

[ref-100] Peterson AT, Papeş M, Soberón J (2008). Rethinking receiver operating characteristic analysis applications in ecological niche modeling. Ecological Modelling.

[ref-101] Peterson A, Soberón J (2012). Species distribution modeling and ecological niche modeling: getting the concepts right. Natureza e Conservação.

[ref-102] Phillips SJ, Anderson RP, Schapire RE (2006). Maximum entropy modeling of species geographic distributions. Ecological Modelling.

[ref-103] Puppato S, Grassi A, Pedrazzoli F, De Cristofaro A, Ioriatti C (2020). First report of *Leptopilina japonica* in Europe. Insects.

[ref-104] QGIS Geographic Information System (2022). QGIS geographic information system. Open Source Geospatial Foundation Project. http://qgis.osgeo.org.

[ref-105] Qiao H, Escobar LE, Peterson AT (2017). Accessible areas in ecological niche comparisons of invasive species: recognized but still overlooked. Scientific Reports.

[ref-106] Quiner CA, Nakazawa Y (2017). Ecological niche modeling to determine potential niche of Vaccinia virus: a case only study. International Journal of Health Geographics.

[ref-107] Raghavan RK, Peterson AT, Cobos ME, Ganta R, Foley D (2019). Current and future distribution of the lone star tick. PLOS ONE.

[ref-108] Ratto F, Bruce T, Chipabika G, Mwamakamba S, Mkandawire R, Khan Z, Mkindi A, Pittchar J, Sallu SM, Whitfield S, Wilson K, Sait SM (2022). Biological control interventions reduce pest abundance and crop damage while maintaining natural enemies in sub-Saharan Africa: a meta-analysis. Proceedings of the Royal Society B.

[ref-109] Raxworthy CJ, Ingram CM, Rabibisoa N, Pearson RG (2007). Applications of ecological niche modeling for species delimitation: a review and empirical evaluation using day geckos (*Phelsuma*) from Madagascar. Systematic Biology.

[ref-110] Rendon D, Hamby KA, Arsenault-Benoit AL, Taylor CM, Evans RK, Roubos CR, Sial AA, Rogers M, Petran A, Van Timmeren S, Fanning P, Isaacs R, Walton V (2020). Mulching as a cultural control strategy for *Drosophila suzukii* in blueberry. Pest Management Science.

[ref-111] Reyes JA, Lira-Noriega A (2020). Current and future global potential distribution of the fruit fly *Drosophila suzukii* (Diptera: Drosophilidae). The Canadian Entomologist.

[ref-112] Robertson MP, Kriticos DJ, Zachariades C (2008). Climate matching techniques to narrow the search for biological control agents. Biological Control.

[ref-113] Rossi Stacconi MV, Buffington M, Daane KM, Dalton DT, Grassi A, Kaçar G, Miller B, Miller JC, Baser N, Ioriatti C, Walton VM, Wiman NG, Wang X, Anfora G (2015). Host stage preference, efficacy and fecundity of parasitoids attacking *Drosophila suzukii* in newly invaded areas. Biological Control.

[ref-114] Rota-Stabelli O, Blaxter M, Anfora G (2013). Drosophila suzukii. Current Biology.

[ref-115] Rotenberry JT, Preston KL, Knick ST (2006). Gis-based niche modeling for mapping species’ habitat. Ecology.

[ref-116] Santoiemma G, Trivellato F, Caloi V, Mori N, Marini L (2019). Habitat preference of *Drosophila suzukii* across heterogeneous landscapes. Journal of Pest Science.

[ref-117] Santos LA dos, Mendes MF, Krüger AP, Blauth ML, Gottschalk MS, Garcia FRM (2017). Global potential distribution of *Drosophila suzukii* (Diptera, Drosophilidae). PLOS ONE.

[ref-118] Savary S, Bregaglio S, Willocquet L, Gustafson D, Mason D’Croz D, Sparks A, Castilla N, Djurle A, Allinne C, Sharma M, Rossi V, Amorim L, Bergamin A, Yuen J, Esker P, McRoberts N, Avelino J, Duveiller E, Koo J, Garrett K (2017). Crop health and its global impacts on the components of food security. Food Security.

[ref-119] Savary S, Willocquet L, Pethybridge SJ, Esker P, McRoberts N, Nelson A (2019). The global burden of pathogens and pests on major food crops. Nature Ecology & Evolution.

[ref-120] Schetelig MF, Lee K-Z, Otto S, Talmann L, Stökl J, Degenkolb T, Vilcinskas A, Halitschke R (2018). Environmentally sustainable pest control options for *Drosophila suzukii*. Journal of Applied Entomology.

[ref-121] Schöneberg T, English LA, Popp J, Hamby KA (2022). Impact of modified caneberry trellis systems on microclimate and habitat suitability for *Drosophila suzukii* (Diptera: Drosophilidae). Journal of Economic Entomology.

[ref-122] Schulz AN, Lucardi RD, Marsico TD (2019). Successful invasions and failed biocontrol: the role of antagonistic species interactions. BioScience.

[ref-123] Shawer R, Garcia FRM (2020). Chemical control of *Drosophila suzukii*. Drosophila suzukii *Management*.

[ref-124] Shawer R, Tonina L, Tirello P, Duso C, Mori N (2018). Laboratory and field trials to identify effective chemical control strategies for integrated management of *Drosophila suzukii* in European cherry orchards. Crop Protection.

[ref-125] Sillero N, Arenas-Castro S, Enriquez-Urzelai U, Vale CG, Sousa-Guedes D, Martínez-Freiría F, Real R, Barbosa AM (2021). Want to model a species niche? a step-by-step guideline on correlative ecological niche modelling. Ecological Modelling.

[ref-126] Simões MVP, Peterson AT (2018). Importance of biotic predictors in estimation of potential invasive areas: the example of the tortoise beetle *Eurypedus nigrosignatus*, in Hispaniola. PeerJ.

[ref-127] Soberón J, Peterson AT (2005). Interpretation of models of fundamental ecological niches and species’ distributional areas. Biodiversity Informatics.

[ref-128] Sun Y, Brönnimann O, Roderick GK, Poltavsky A, Lommen STE, Müller-Schärer H (2017). Climatic suitability ranking of biological control candidates: a biogeographic approach for ragweed management in Europe. Ecosphere.

[ref-129] Tabachnick B, Fidell LS (2007). Using multivariate statistics.

[ref-130] Tait G, Mermer S, Stockton D, Lee J, Avosani S, Abrieux A, Anfora G, Beers E, Biondi A, Burrack H, Cha D, Chiu JC, Choi M-Y, Cloonan K, Crava CM, Daane KM, Dalton DT, Diepenbrock L, Fanning P, Ganjisaffar F, Gómez MI, Gut L, Grassi A, Hamby K, Hoelmer KA, Ioriatti C, Isaacs R, Klick J, Kraft L, Loeb G, Rossi-Stacconi MV, Nieri R, Pfab F, Puppato S, Rendon D, Renkema J, Rodriguez-Saona C, Rogers M, Sassù F, Schöneberg T, Scott MJ, Seagraves M, Sial A, Van Timmeren S, Wallingford A, Wang X, Yeh DA, Zalom FG, Walton VM (2021). *Drosophila suzukii* (Diptera: Drosophilidae): a decade of research towards a sustainable integrated pest management program. Journal of Economic Entomology.

[ref-131] Tepa-Yotto GT, Gouwakinnou GN, Fagbohoun JR, Tamò M, Saethre M-G (2021a). Horizon scanning to assess the bioclimatic potential for the alien species *Spodoptera eridania* and its parasitoids after pest detection in West and Central Africa. Pest Management Science.

[ref-132] Tepa-Yotto GT, Tonnang HEZ, Goergen G, Subramanian S, Kimathi E, Abdel-Rahman EM, Flø D, Thunes KH, Fiaboe KKM, Niassy S, Bruce A, Mohamed SA, Tamò M, Ekesi S, Sæthre M-G (2021b). Global habitat suitability of *Spodoptera frugiperda* (JE Smith) (Lepidoptera, Noctuidae): key parasitoids considered for its biological control. Insects.

[ref-133] Tocchio LJ, Gurgel-Gonçalves R, Escobar LE, Peterson AT (2015). Niche similarities among white-eared opossums (Mammalia, Didelphidae): is ecological niche modelling relevant to setting species limits?. Zoologica Scripta.

[ref-134] Tuszynski J (2021). caTools: tools: moving window statistics, GIF, Base64, ROC AUC, *etc* (see https://CRAN.R-project.org/). https://CRAN.R-project.org/.

[ref-135] Valencia-Rodríguez D, Jiménez-Segura L, Rogéliz CA, Parra JL (2021). Ecological niche modeling as an effective tool to predict the distribution of freshwater organisms: the case of the Sabaleta *Brycon henni* (Eigenmann, 1913). PLOS ONE.

[ref-136] Van Driesche RG, Hoddle M (1997). Should arthropod parasitoids and predators be subject to host range testing when used as biological control agents?. Agriculture and Human Values.

[ref-137] Van Lenteren JC (2012). The state of commercial augmentative biological control: plenty of natural enemies, but a frustrating lack of uptake. BioControl.

[ref-138] Van Timmeren S, Isaacs R (2013). Control of spotted wing *Drosophila*, *Drosophila suzukii*, by specific insecticides and by conventional and organic crop protection programs. Crop Protection.

[ref-139] Vreysen MJB, Robinson AS, Hendrichs J, Kenmore P, Vreysen MJB, Robinson AS, Hendrichs J (2007). Area-wide integrated pest management (AW-IPM): principles, practice and prospects. Area-wide control of insect pests.

[ref-140] Walsh DB, Bolda MP, Goodhue RE, Dreves AJ, Lee J, Bruck DJ, Walton VM, O’Neal SD, Zalom FG (2011). *Drosophila suzukii* (Diptera: Drosophilidae): invasive pest of ripening soft fruit expanding its geographic range and damage potential. Journal of Integrated Pest Management.

[ref-141] Wan J, Qi G, Ma J, Ren Y, Wang R, McKirdy S (2020). Predicting the potential geographic distribution of *Bactrocera bryoniae* and *Bactrocera neohumeralis* (Diptera: Tephritidae) in China using MaxEnt ecological niche modeling. Journal of Integrative Agriculture.

[ref-142] Wang J, Zheng Y, Fan L, Wang W (2022). Surveys of *Drosophila suzukii* (Diptera: Drosophilidae) and its host fruits and associated parasitoids in northeastern China. Insects.

[ref-143] Wang X, Daane KM, Hoelmer KA, Lee JC, Garcia FRM (2020). Biological control of spotted-wing *Drosophila*: an update on promising agents. Drosophila suzukii management.

[ref-144] Wang X, Hogg BN, Hougardy E, Nance AH, Daane KM (2019). Potential competitive outcomes among three solitary larval endoparasitoids as candidate agents for classical biological control of *Drosophila suzukii*. Biological Control.

[ref-145] Warren DL, Glor RE, Turelli M (2008). Environmental niche equivalency versus conservatism: quantitative approaches to niche evolution. Evolution.

[ref-146] Warren DL, Matzke NJ, Cardillo M, Baumgartner JB, Beaumont LJ, Turelli M, Glor RE, Huron NA, Simões M, Iglesias TL, Piquet JC, Dinnage R (2021). ENMTools 1.0: an R package for comparative ecological biogeography. Ecography.

[ref-147] Warren DL, Seifert SN (2011). Ecological niche modeling in Maxent: the importance of model complexity and the performance of model selection criteria. Ecological Applications.

[ref-148] Yeh DA, Drummond FA, Gómez MI, Fan X (2020). The economic impacts and management of spotted wing *Drosophila* (*Drosophila suzukii*): the case of wild blueberries in Maine. Journal of Economic Entomology.

