# Supplementary figures and images for: Mapping the global distribution of invasive pest Drosophila suzukii and parasitoid Leptopilina japonica: implications for biological control
Source: PeerJ. 2023 Apr 24;11:e15222. doi: 10.7717/peerj.15222 (PMC10135410; doi:10.7717/peerj.15222)

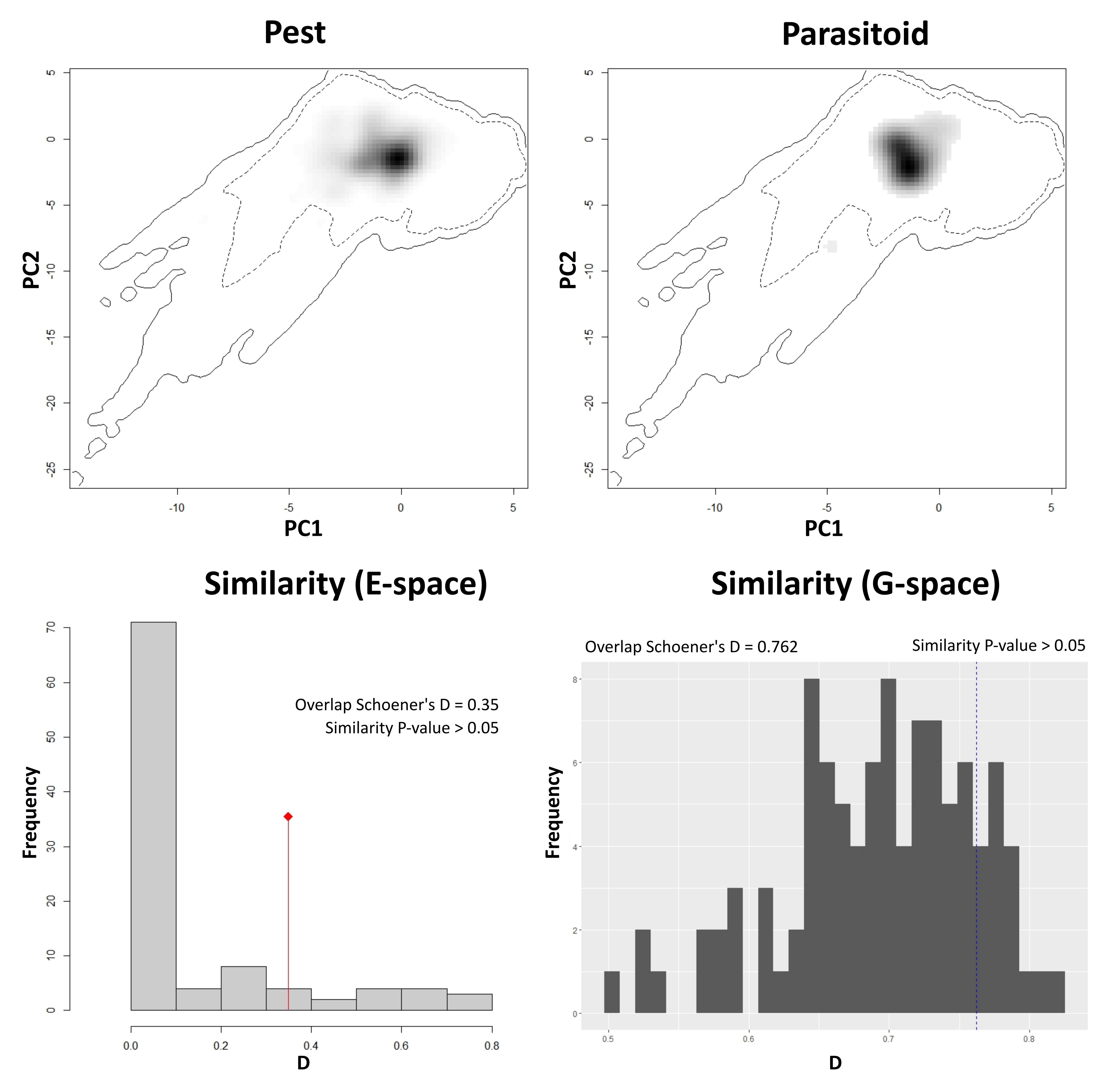

Supplement: File S4 — Niches of pest and parasitoid are represented along the first two principal components (top panel). Dark shading indicates the occurrence densities of pest and parasitoid by cell. The solid and dashed contour lines indicate 100% and 50% of background environment in the area M. The empirical D value fell within the null distribution of D values generated in both tests (Broennimann et al., 2012, bottom left; Warren, Glor & Turelli, 2008, bottom right), indicating the non-rejection of the null hypothesis of niche similarity. E-space: Environmental space, G-space: Geographic space [file peerj-11-15222-s004.png]
